# Early overyielding in a mixed deciduous forest is driven by both above- and below-ground species-specific acclimatization

**DOI:** 10.1093/aob/mcae150

**Published:** 2024-09-23

**Authors:** Ramona Werner, Lisa T Gasser, Matthias Steinparzer, Mathias Mayer, Iftekhar U Ahmed, Hans Sandén, Douglas L Godbold, Boris Rewald

**Affiliations:** Institute of Forest Ecology, Department of Forest and Sciences, University of Natural Resources and Life Sciences (BOKU), Gregor-Mendel-Straße 33, 1190 Vienna, Austria; Institute of Forest Ecology, Department of Forest and Sciences, University of Natural Resources and Life Sciences (BOKU), Gregor-Mendel-Straße 33, 1190 Vienna, Austria; Institute of Forest Ecology, Department of Forest and Sciences, University of Natural Resources and Life Sciences (BOKU), Gregor-Mendel-Straße 33, 1190 Vienna, Austria; Institute of Forest Ecology, Department of Forest and Sciences, University of Natural Resources and Life Sciences (BOKU), Gregor-Mendel-Straße 33, 1190 Vienna, Austria; Forest Soils and Biogeochemistry, Swiss Federal Institute for Forest, Snow and Landscape Research (WSL), 8903 Birmensdorf, Switzerland; Institute of Forest Ecology, Department of Forest and Sciences, University of Natural Resources and Life Sciences (BOKU), Gregor-Mendel-Straße 33, 1190 Vienna, Austria; Federal Research and Training Center for Forests (BFW), Department of Forest Protection, Seckendorff-Gudent-Weg 8, 1131 Vienna, Austria; Institute of Forest Ecology, Department of Forest and Sciences, University of Natural Resources and Life Sciences (BOKU), Gregor-Mendel-Straße 33, 1190 Vienna, Austria; Institute of Forest Ecology, Department of Forest and Sciences, University of Natural Resources and Life Sciences (BOKU), Gregor-Mendel-Straße 33, 1190 Vienna, Austria; Mendel University in Brno, Faculty of Forestry and Wood Technology, Department of Forest Protection and Wildlife Management, Zemědělská 3, 61300 Brno, Czech Republic; Institute of Forest Ecology, Department of Forest and Sciences, University of Natural Resources and Life Sciences (BOKU), Gregor-Mendel-Straße 33, 1190 Vienna, Austria; Mendel University in Brno, Faculty of Forestry and Wood Technology, Department of Forest Protection and Wildlife Management, Zemědělská 3, 61300 Brno, Czech Republic; Vienna Scientific Instruments, Heiligenkreuzer Straße 433, 2534 Alland, Austria

**Keywords:** Mixed plantations, tree diversity, biomass allocation, canopy, interspecific competition, overyielding, plasticity, fine roots, *Acer platanoides*, *Tilia cordata*, *Quercus robur*, *Carpinus betulus*

## Abstract

**Background and Aims:**

Mixed forest plantations are increasingly recognized for their role in mitigating the impacts of climate change and enhancing ecosystem resilience. Yet, there remains a significant gap in understanding the early-stage dynamics of species trait diversity and interspecies interactions, particularly in pure deciduous mixtures. This study aims to explore the timing and mechanisms by which trait diversity of deciduous species and competitive interactions influence yield, carbon allocation and space occupation in mixed forests, both above and below ground.

**Methods:**

A forest inventory was conducted in planted monocultures, two-species and four-species mixtures of European *Acer*, *Tilia*, *Carpinus* and *Quercus*, representing a spectrum from acquisitive to conservative tree species. Effects of competition were assessed with linear mixed-effects models at the level of biomass and space acquisition, including leaf, canopy, stem and fine root traits.

**Key Results:**

Early above-ground growth effects were observed 6 years post-planting, with significant biomass accumulation after 8 years, strongly influenced by species composition. Mixtures, especially with acquisitive species, exhibited above-ground overyielding, 1.5–1.9 times higher than monocultures. Fine roots showed substantial overyielding in high-diversity stands. Biomass allocation was species specific and varied markedly by tree size and the level of diversity and between acquisitive *Acer* and the more conservative species. No root segregation was found.

**Conclusions:**

Our findings underscore the crucial role of species trait diversity in enhancing productivity in mixed deciduous forest plantations. Allometric changes highlight the need to differentiate between (active) acclimatizations and (passive) tree size-related changes, but illustrate major consequences of competitive interactions for the functional relationship between leaves, stem and roots. This study points towards the significant contributions of both above- and below-ground components to overall productivity of planted mixed-species forests.

## INTRODUCTION

Tree planting as a key measure to mitigate anthropogenic CO_2_ emissions (Bastin *et al.*, 2019) is underscored by the European Commission’s ambitious ‘3 Billion Trees Pledge’ under the European Green Deal (European Commission, 2022). In contrast to ‘traditional’ monoculture plantations, often considered vulnerable to drought and disturbance (Bauhus *et al.*, 2017; Jactel *et al.*, 2017), mixed forest stands have been proposed as a potential solution to address the challenges posed by climate change and the biodiversity crisis (FAO, 2023). Multispecies forests hold several benefits in comparison to monocultures, including increased carbon sequestration (Liu *et al.*, 2018; Xiang *et al.*, 2022), improved resistance and/or resilience to pests and droughts (Griess and Knoke, 2011; Jactel *et al.*, 2017; Liu *et al.*, 2018), and providing habitat for a more diverse set of species (Felton *et al.*, 2010; Cavard *et al.*, 2011). As forest monocultures are increasingly transformed into ‘climate-smart’ mixed forests, especially in temperate zones (Palacios-Agundez *et al.*, 2015; Huang *et al.*, 2018; Liu *et al.*, 2018), understanding their diversity–productivity relationships and the underlying changes in species-level traits is key to predict ecosystem functioning.

Plant individuals are typically surrounded by others, leading to intense interactions among neighbouring plants. Negative interactions often result from the depletion of resources (Grace and Tilman, 1990). Although positive effects on growth or survival (i.e. facilitation) have been reported even for tree seedling monocultures, particularly in more resource-limited environments (Fajardo and McIntire, 2011), complementary resource/space use above and below ground is thought to explain most of the increased productivity in temperate forest mixtures (Pretzsch and Schütze, 2009, 2016; van de Peer *et al.*, 2017*a*). This greater community productivity, in comparison to average monocultures in similar environments, is known as ‘overyielding’ (Pretzsch *et al.*, 2013; Göransson *et al.*, 2016; Ammer, 2019; Lwila *et al.*, 2021). For example, intraspecific competition in monocultures might limit crown expansion (Pretzsch and Schütze, 2016), whereas mixed-species stands might allow for a complementary, greater space utilization through intrinsic differences in species phenologies and/or plastic acclimatization of tree growth patterns (Seidel *et al.*, 2011; Barbeito *et al.*, 2014; Martin-Blangy *et al.*, 2023). Niche differentiation, such as canopy stratification, has been shown to increase overall light interception (Williams *et al.*, 2017; Forrester *et al.*, 2018). Likewise, root stratification and/or complementary root physiology/interactions with mycorrhizal symbionts might increase below-ground resource acquisition in mixed stands (Rewald and Leuschner, 2009*b*; Lwila *et al.*, 2021; Kinzinger *et al.*, 2024). However, niche partitioning is notoriously difficult to study and is primarily inferred from growth pattern or trait proxies (Silvertown, 2004). Yet, (mechanical) interactions, such as twig abrasion, in addition to (kin recognition by) exudates/volatiles and indirect effects of herbivores and pathogens (Frech *et al.*, 2003; Pierik *et al.*, 2013; Bauhus *et al.*, 2017; Mazal *et al.*, 2023) can influence stand dynamics and might thus weaken the relationship between niche differentiation and performance. Furthermore, overyielding has been demonstrated *in situ* with a focus on above-ground production (Pretzsch and Schütze, 2009; van de Peer *et al.*, 2017*a*; Lu *et al.*, 2018; Dietrich *et al.*, 2023), although investigations of diversity effects on biomass allocation to and phenotypic plasticity of tree fine root systems are generally rare (Jacob *et al.*, 2013; Domisch *et al.*, 2015; Fruleux *et al.*, 2018; Shu *et al.*, 2018). This gap, as recently highlighted by Jacobsen (2023), is notable given the significant impact that rooting patterns have on biogeochemical cycles and resource acquisition (Gobran *et al.*, 1998; Maeght *et al.*, 2013).

An expanded species portfolio in planted forests is generally considered to be an insurance against an uncertain future under climate change, because it provides more ‘opportunities’ to respond to stress (Bolte *et al.*, 2009; Keenan, 2015; Blondeel *et al.*, 2024). Community overyielding does not require all species to contribute equally, but (more) productive plant species might over-compensate for reduced growth and/or neutral responses of others (Steinparzer *et al.*, 2022; Urgoiti *et al.*, 2023*b*). The competitive ability and fitness of a plant in given environmental conditions is strongly related to its functional traits; species with similar traits might compete in a similar manner for growing space and resources (Forrester, 2014; Fichtner *et al.*, 2017). In general, mixtures of trees with complementary structural and/or functional traits, i.e. fast-growing (‘acquisitive’) species with slow-growing (‘conservative’) species, have thus been proposed to increase forest productivity (Griess and Knoke, 2011; Jacob *et al.*, 2013; Fichtner *et al.*, 2017; Liu *et al.*, 2018). Tree species with ‘acquisitive’ traits are characterized by rapid resource acquisition and lower wood densities, whereas ‘conservative’ species tend to have slower resource acquisition and growth rates, a lower specific leaf area and nutrient content, but often higher structural investment, with greater wood densities and longer organ life spans (Reich, 2014; Gorné *et al.*, 2022).

In contrast to natural regeneration of saplings under an overstorey (e.g. Brandeis *et al.*, 2001), space above and below ground is largely unoccupied in the initial years after tree planting, and direct competitive interactions among tree seedlings are thought to be minimal, favouring the rapid growth of ‘acquisitive’ species (Ricard *et al.*, 2003; but for competition with ground vegetation, see e.g. Davis *et al.*, 1998). Around early canopy closure, it can be assumed that acquisitive species, with their rapid height growth and extensive crown expansion, pre-empt light. This advantage can (initially) hamper the growth of (yet) less tall, more ‘conservative’ species. Consequently, acquisitive species might dominate the community yield at early stages (Urgoiti *et al.*, 2023*a*, 2023*b*). However, positive biodiversity–productivity relationships tend to strengthen over time (Thurm and Pretzsch, 2016; Tatsumi, 2020; Dietrich *et al.*, 2023), although for many deciduous mixtures, it remains unclear at what point interspecific interactions significantly affect productivity. In the absence of spatial/temporal heterogeneity, natural enemies or stand management, insufficient resource partitioning might cause a gradual species exclusion over succession (Petrovska *et al.*, 2021). However, the impact of diversity on survival rates during stand establishment remains uncertain, with both negative and neutral effects being reported (Searle *et al.*, 2022; Blondeel *et al.*, 2024).

Although species versus (functional) trait diversity effects on competitive outcomes have long been debated in community ecology (e.g. Cadotte *et al.*, 2011), it is becoming increasingly clear that (positive) productivity incentives strongly depend on the functional diversity within mixed forests (Jacob *et al.*, 2013; Domisch *et al.*, 2015; Schuster *et al.*, 2023). However, through plasticity, trees adapt traits and trait syndromes to above- and below-ground resource availability, as determined by spatiotemporal environmental conditions and competitors (Hofhansl *et al.*, 2021; Weithmann *et al.*, 2022). The response norms, i.e. the direction and degree of this plasticity, are highly genotype dependent (Wortemann *et al.*, 2011; Cope *et al.*, 2021) and modulated by the availability of resources (Hautier *et al.*, 2009; Rewald and Leuschner, 2009*b*; Poorter *et al.*, 2012). Resource competition can lead to a (concurrent) acclimatization at different plant organizational levels (Grams and Andersen, 2007), such as changes in physiology, morphology (e.g. specific root area), size (e.g. tree height) and/or the relationship between organs (e.g. leaf area to root area ratio, mass fractions). Although the development of an integrated framework for trait coordination above and below ground has recently gained increasing attention (Weigelt *et al.*, 2021), it remains largely unclear whether and how different (tree) species ‘coordinate’ trait plasticity across organs under competition. Plant carbon (C) allocation has been described in terms of optimization, economic theory or trade-offs between C investment and return of resources (e.g. Franklin *et al.*, 2012). Carbon allocation has been reported to scale with size in mature trees and seedlings (e.g. Niklas and Enquist, 2002), with growth being largely affected by the availability of nutrients, water or temperature (Prescott *et al.*, 2020). Whatever the (dominant) mechanisms, environmental factors, including competitive situations, affect plant C allocation (Millard *et al.*, 2007; Poorter *et al.*, 2012; Sun *et al.*, 2020). Although trait acclimatization and biomass allocation patterns might thus provide valuable insights into plant functioning and the mechanisms driving community productivity (Poorter *et al.*, 2012; Lübbe *et al.*, 2017), information on the effects of interspecific competitors on tree traits and allocation above and particularly below ground remain scarce, even for key species identified as suitable candidates for future, mixed ‘climate-smart’ forests (Leuschner *et al.*, 2024).

In this study, we selected a portfolio of four tree species typical of the Central European upland (colline) vegetation zone, with different life-history strategies along an acquisitive–conservative gradient, to investigate the dynamics of growth and survival during early stand development, yield effects of mixtures above and below ground, and responses of individual species to allospecific neighbours at the level of biomass allocation and canopy and rooting space acquisition. *Acer platanoides* is a fast-growing, acquisitive species (Caudullo and de Rigo, 2016), and *Tilia cordata* and *Carpinus betulus* are species considered to have intermediate acquisitive and conservative traits with average growth rates (Eaton *et al.*, 2016*a*; Sikkema *et al.*, 2016). *Quercus robur* is a shade-intolerant, slow growing, conservative climax species with moderate drought tolerance (Eaton *et al.*, 2016*b*). We monitored survival and growth 3, 6 and 8 years after planting and determined above- and below-ground biomass and space utilization after canopy closure to gain a better understanding of how tree diversity can influence species-specific niche construction and thus the mechanisms leading to overyielding at the stand level, with implications for both ecological theory and the management of mixed forests. We hypothesized that:

(1) Yield benefits of planted mixed forests occur as early as canopy closure and are based on the acquisitive species in the portfolio.
(2) Species in mixed stands possess yielding effects not only at the level of wood biomass, but also at the level of leaves and fine roots, translating above ground into overyielding effects at stand level.
(3) Plastic allocation of biomass across tree organs and architectural traits, shaped by diversity levels, drives distinct, species-specific and independent patterns in above- and below-ground space utilization.

## MATERIALS AND METHODS

### Study site and experimental set-up

**Fig. 1.**
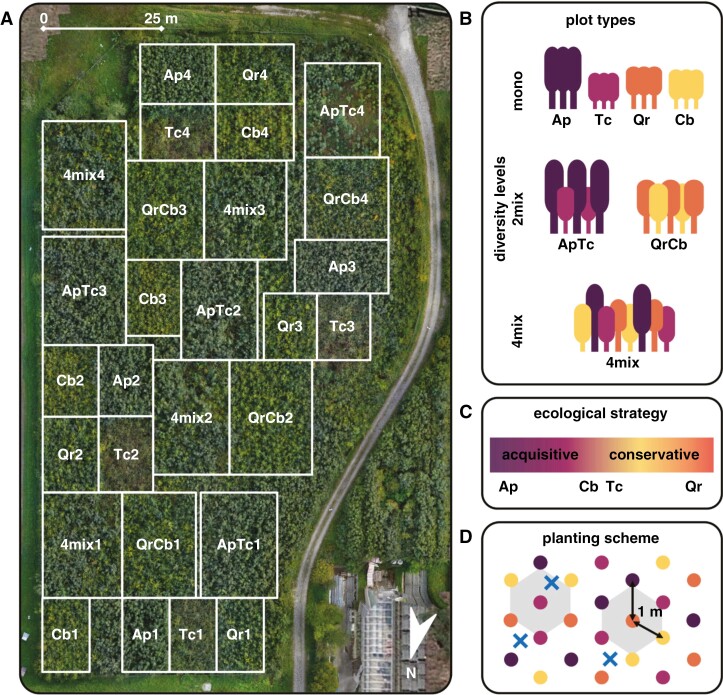
(A) Aerial map of the B-Tree experiment (2021). Ap = *Acer platanoides*, ApTc = mixture of Ap and Tc, Cb = *Carpinus betulus*, Qr = *Quercus robur*, QrCb = mixture of Qr and Cb, Tc = *Tilia cordata*, 4mix = four-species mixture; trailing numbers indicate blocks. (B) Seven plot types at three diversity levels. (C) Ecological strategies of trees (simplified). (D) Planting scheme of a typical 4mix section. Different species are represented by different colours. Trees have been planted 1 m apart, creating a hexagonal pattern (grey); each tree has six neighbours. Typical root sampling locations between a triplet of trees are marked with a blue ‘×’.

This study was conducted at the B-Tree experimental site in Tulln an der Donau as part of the tree diversity network ‘TreeDivNet’ (Verheyen *et al.*, 2016). The site is located in eastern Austria (48°19ʹ2.989″N, 16°4ʹ0.613″E) and covers an area of ~1.2 ha. The mean annual temperature is 10.5 °C, and the mean annual precipitation is 657 mm, with frequent dry spells in spring and/or summer. For additional meteorological information, see Supplementary Data Fig. S1 and Supplementary Information Supplementary Data Detailed Site Information. The soil type is moist Chernozem; the soil is hydromorphic, humus-rich and contains free calcium carbonate, originating from Danube sediments. The soil has a pH_(H2O)_ of 8.28 ± 0.01, and a C to nitrogen ratio of 19.7 ± 0.7 in the topsoil (0–20 cm). A thin (<1–2 mm) layer of organic material (Litter and Fermentation Horizon) developed on top of the mineral soil during the experiment. For more details on soil properties, see Supplementary Information Supplementary Data Detailed Site Information. The former land use was grassland, with sparse tree and shrub cover (Supplementary Data Fig. S2); the site was cleared in 2012. In 2013, ~12 000 2-year-old trees (Murauer Forstpflanzen, Orth im Innkreis, Austria), including maple (*Acer platanoides* L.; Ap), lime (linden, basswood; *Tilia cordata* Mill.; Tc), hornbeam (*Carpinus betulus* L.; Cb) and oak (*Quercus robur* L.; Qr), were planted at three diversity levels: monocultures of all four species, two variants of two-species mixtures (2mix; ApTc and QrCb) and four-species mixtures (4mix), resulting in seven different plot types (Fig. 1B). The species portfolio includes deciduous broadleaf trees typical of Central European colline vegetation zones, which differ in their resource acquisition and use strategies, ranging from highly acquisitive (Ap) to moderately acquisitive/more conservative species (Cb and Tc) to highly conservative (Qr) (Caudullo and de Rigo, 2016; Eaton *et al.*, 2016*a*, 2016*b*; Sikkema *et al.*, 2016; Leuschner and Ellenberg, 2018). All four species have been suggested to be suitable timber species for the Central European Forestry Sector in a drier and warmer future climate (Leuschner *et al.*, 2024). The design created plots with preferred ectomycorrhizal (EM) host trees (Qr and Cb) versus strict (Ap) or potentially opportunistic (Tc) arbuscular mycorrhizal (AM) host trees (Dudka *et al.*, 2023). Results for individual species in mixtures are indicated by adding the diversity level to the species abbreviation, e.g. Ap_mono_ for Ap trees in monocultures, Ap_2mix_ in two-species and Ap_4mix_ in four-species mixtures.

A total of 28 plots were established (Fig. 1A), with the seven plot types being replicated in four blocks distributed across the site. Seedlings were planted in offset (staggered) rows to achieve a uniform spacing of 1 m between trees (i.e. 1 m within rows, 0.87 m between rows; Fig. 1D). Thus, each tree has six neighbours; the spatial distribution of tree species in the mixtures is irregular, resulting in different neighbourhood situations characterized by different numbers of conspecific or heterospecific neighbours (Fig. 1D). The size of the plots varies between ~131 and ~ 313 m^2^ in monocultures and mixtures, respectively, to allow sufficient replication (≥150, ≥180 and ≥90 tree individuals per species and plot in monocultures, two-species and four-species mixtures, respectively). Within the mixtures, the individual tree species were planted in equal numbers, i.e. 50 % each in 2mix and 25 % each in 4mix plots. Within the first two growing seasons (i.e. until autumn 2014), dead seedlings were replaced with trees of the same age and size as the surviving seedlings at the time of replacement, all sourced from the same nursery and seed source. As a result, all the trees in the experiment are the same age, despite replanting. To reduce microclimatic/edge effects, the space between plots was planted with tree mixtures following the same planting pattern. Dedicated footpaths were created on plot borders to reduce trampling effects on the plots.

To minimize the effect of adjacent plots and edges, the two outer rows per plot were removed from all data analyses. In addition, for two-species and four-species mixtures, tree individuals with a majority of conspecific neighbours (four to six of six) were excluded because they did not (by chance) resemble the character of the respective mixture. Plots ApTc4, Cb4 and Tc4 were excluded from this dataset because canopy closure was not complete by 2021, and the majority of trees were still in a shrub-like stage (Fig. 1A; Supplementary Data Fig. S2), potentially owing to a greater soil density and less favourable water supply conditions in this part of the experimental site (data not shown).

### Tree survival, growth and allometric equations

Measurements were taken in April/May 2016, May/June 2019 and March 2021 to determine height (H; in metres), stem diameter (in centimetres) and tree mortality in all plots. Tree height and diameter were determined manually using telescopic measuring rods and callipers; total tree height was measured as the distance between ground level and the shoot height along the stem axis (West, 2015). Stem diameter was measured at 17.5 cm in 2016 and 2019, and at 17.5 cm and 130 cm (diameter at breast height; DBH) in 2021; two measurements were conducted 90° shifted using digital callipers; measurements were averaged for further analyses. We categorized trees as dead following the approach of Vanderwel *et al.* (2006), using the following criteria: absence of new leaves or buds; lack of foliage; inflexibility of branches or trunks; and absence in the planting row. The mortality rate (as a percentage) was expressed as a cumulative value for the subsequent inventory years. Height (in metres) and basal area (BA_17.5_; in centimetres squared) at 17.5 cm stem height of individual living trees and plot averages were calculated for 2016, 2019 and 2021; wood biomass (WB; in tons per hectare) and basal area at breast height (130 cm; BA_130_; in centimetres squared per hectare) per plot and species, i.e. including dead individuals, were calculated for the year 2021. Allometric models for stem and branch biomass (wood biomass; WB) were established in June 2021 by harvesting ten trees per species (covering the full size range observed in the plots) from a surplus area planted in adjacence to the experimental site, following Ahmed *et al.* (2019).

Allometric models, developed by using tree variables (i.e. basal diameter, DBH and height) were evaluated through ‘goodness of fit’ and performance statistics including coefficient of determination (*R*^2^), root mean square error (RMSE) and the corrected Akaike information criterion (AICc). Models are given in Supplementary Data Table S1.

To determine potential over- or underyielding effects, the expected total biomass and biomass per compartment, i.e. leaf, WB and root biomasses (see below) of the mixtures (in tons per hectare) were determined based on the observed average biomasses (total or per organ) of the respective monocultures and compared with the observed values in mixtures (Pretzsch and Schütze, 2009). We investigated the relationship between total, leaf and/or fine root biomasses (see below) with linear models. Biomasses were normalized to the species proportions in diversity levels (100–25 %; hereafter indicated as normalized biomass with a subscript ‘n’; Pretzsch *et al.*, 2013). Normalized total biomass (TB_n_ = LB_n_, + WB_n_) was related to LB_n_ or FRB_n_ using a log_10_ scale.

### Leaf biomass, leaf area and crown dimensions

To determine leaf biomass (LB) and leaf area index (LAI), 128 leaf litter traps were deployed in 2021/22, following Ahmed *et al.* (2019). In the monoculture plots, three traps were randomly distributed in their centres, equidistant to individual trees. In the mixtures, five traps were placed similarly, to ensure an approximately equal influence of each present tree species; leaves were collected biweekly during the main abscission period and monthly thereafter. Leaf litter was sorted by tree species for each plot, dried (80 °C for 48 h) and weighed (±0.001 g); LB was calculated at both tree species (in tons per hectare) and plot level (in tons per hectare). Specific leaf area (SLA) was calculated by scanning 100 freshly fallen leaves per species and plot in autumn 2021 with a flatbed scanner (EPSON Expression 10000 XL; 300 dpi, colour) and analysing their surface area using WinFolia software (Pro 2014a 32-bit; Regent Instruments, Quebec, Canada), followed by drying (80 °C for 48 h) and weighing. The SLA was calculated by dividing the leaf area (in centimetres squared) per dry mass (in grams) (Pérez-Harguindeguy *et al.*, 2013). To determine the total and species-specific leaf area indices (LAI; in metres squared per metre squared) per plot, the SLA was multiplied by the litter dry mass and divided by the area covered by litter traps. To compare monocultures and mixtures at the species level, the LAI values were normalized based on the mixture-specific proportions of each species in 2mix and 4mix plots (LAI_n_; Pretzsch *et al.*, 2013). Crown dimension, including height of the first living branch with a diameter ≥1 cm (hFB; in metres), maximum crown diameter (MCD; in metres) and the height of maximum crown diameter (hMCD; in metres), were measured in spring 2021 across 15 trees per species and plot using telescopic measuring rods and tape measures (West, 2015). Species- and community-specific trait values were then calculated.

### Root biomass, root area index and root system segregation

In July 2021, 160 soil cores were collected to a depth of 40 cm using a soil corer (inner diameter of 6.8 cm). Sampling points were located at the mid-point between three trees (‘triplets’; Fig. 1D). For monocultures, four cores were collected per plot. In mixtures, sampling points covered all plot representative triplet combinations of tree species per plot type. Thus, in two-species mixtures, six cores were collected per plot, with three cores taken from each of the two relevant triplet combinations: ‘ApApTc’ and ‘ApTcTc’, or ‘QrQrCb’ and ‘QrCbCb’, respectively. For four-species mixtures, 12 cores were collected per plot, with three cores from each triplet type: ‘ApQrTc’, ‘ApCbTc’, ‘ApCbQr’ and ‘CbQrTc’. The (thin) litter layer was removed prior to sampling. Soil cores were separated on site into upper (0–20 cm) and lower (20–40 cm) soil horizons and stored at 4 °C until processing. Samples were sieved (2 mm), and roots (≥1 cm in length) and stones were collected. Fine roots (diameter, *d* ≤ 2 mm) were rinsed, sorted by species according to morphological criteria (Rewald *et al.*, 2012), and living roots were stored at 4 °C until further processing. Owing to high spatial heterogeneity, coarse root biomass (CRB; *d* > 2 mm) was analysed only at a plot level (in tons per hectare). Dead fine roots were not considered further because they represented <10 % of the total fine root mass (data not shown) and could not be categorized into species. Three random fine root branches per species, sample and depth were used for morphological analysis with WinRhizo (PRO 2012, Regent Instruments, Canada; Epson Expression 10000 XL with transparency unit; 600 dpi, grey scale). All samples were then dried at 40 °C (until weight constancy) and weighed (±0.0001 g). The fine root surface area (in centimetres squared) and weight were used to calculate the specific root area (SRA; in centimetres squared per gram). The fine root area index (RAI; in metres squared per metre squared) per sample location was obtained by multiplying the SRA by the total fine root biomass (FRB; in tons per hectare) and expressed as total and per species values per plot type (Rewald and Leuschner, 2009*a*). As with LAI, normalized RAI values (RAI_n_) were calculated to compare monocultures and mixtures at the species level. To analyse the distribution patterns of fine roots, species-specific relative biomass proportions of the total fine root biomass (in grams per gram) per soil core were calculated, analogous to the root length profiling pattern used earlier (Luo *et al.*, 2021). Fine root distribution expressed as root surface area, suggested as a better indicator for exploitation effort than root mass, did not yield different results (data not shown). The observed root biomass proportions of each species were related to expected, tree abundance-related proportions derived by the surrounding tree individuals (triplet). For instance, at a sample point surrounded by two Ap trees and one Tc tree within a two-species mixture (‘ApApTc’), the expected biomass proportions were allocated as 66 % to Ap and 33 % to Tc. Within four-species mixture plots, a sample point surrounded, e.g. by Ap, Qr and Tc (‘ApQrTc’), was expected to yield biomass proportions of 0.333 for each surrounding species, with Cb contributing none. Frequencies of soil cores containing more, equal or less than expected fine root biomass contributions of a specific species, relative to the total fine root biomass per soil core, were calculated based on the total number of soil cores within a diversity level. The contribution of individual species to FRB per soil core was calculated at the level of species triplet per plot. Root data are displayed either per soil horizon (0–20 or 20–40 cm) or for the total profile (0–40 cm); vertical root segregation was studied by comparing (relative) biomass distribution across horizons.

### Total biomass and mass fractions

Total plant biomass (TB) was calculated as the sum of LB, WB and FRB for species-wise comparisons; CRB was considered only for plot-wise comparisons (Supplementary Data Fig. S3). Mass fractions of leaves (LMF; as a percentage), stems and branches (SMF; as a percentage) and fine roots (RMF; as a percentage) were calculated to determine changes in biomass allocation patterns (Poorter *et al.*, 2012). To study the potential effect of tree size on allocation patterns, we used linear models to analyse the relationship between normalized TB_n_ and LMF, in addition to RMF. Changes in mass fractions (ΔLMF, ΔSMF and ΔRMF) were calculated for mixtures compared with monocultures. Including TB or TB_n_ as covariates in these models gave similar outcomes (data not shown).

The ratios between LB_n_ and diameter at breast height (LB_n_/DBH) and between FRB_n_ and DBH (FRB_n_/DBH) were calculated to assess the coordination between absorbing organs and the stem of a tree, reflecting the modulation of metabolic scaling theory by above-ground factors, such as tree size and canopy position, which influence light interception in mixed stands (Laubhann *et al.*, 2010; Poorter *et al.*, 2015; Gomarasca *et al.*, 2023), and by resource competition below ground. Furthermore, these ratios might serve as indicators of the balance between the transpiring or absorbing organs of trees and the potential conducting area of the stem (Midgley, 2003). Especially in young trees, the stem dimension can serve as a proxy for sapwood area (Raulier *et al.*, 2002). Finally, the leaf area to (fine) root area ratio (LAI/RAI) was calculated, providing a measure for the ratio between absorbing surfaces above and below ground (to 40 cm soil depth). To study a potential systematic variation with tree (stem) size (plot averages), we applied linear models to analyse the relationship between DBH and LAI/RAI, LB_n_ and FRB_n_ (Supplementary Data Fig. S4); similar patterns occurred when testing DBH against LB_n_/DBH and FRB_n_/DBH ratios (data not shown).

### Statistical analysis

Statistical analyses were conducted using R (v.4.3.2, 31 October 2023; R Core Team, 2023) and RStudio (v.2023.09.1; RStudio Team, 2023). Linear mixed-effects models (LMMs) from the ‘nlme’ package (Pinheiro and Bates, 2023) were used to investigate the effects of mixed tree plantations on three levels: diversity (i.e. mono, 2mix and 4mix), plot type (i.e. Ap, Tc, Qr, Cb, ApTc, QrCb and 4mix) and individual tree species (i.e. Ap, Tc, Qr and Cb). Blocks (i.e. 1–4) and/or individual plots were used as nested random effects. For each separate model, we tested whether inclusion of the variable ‘block’ had a significant effect. If this was not the case, only the individual plots were used as a random effect. Temporal analyses included the tree individual, and analyses of root distribution patterns included the individual soil core as a random effect. Response variables were the aspects of growth performance (basal area), mortality, biomass, traits such as crown shape, LAI, RAI, and calculated ratios, in addition to root distribution patterns. Pairwise comparisons were analysed using the ‘lsmeans’ package (Lenth, 2016), with Tukey’s *P*-value adjustment.

Analyses of growth, based on BA_17.5_, were performed at the level of individual trees within plots; dead individuals were excluded from this analysis. Mortality rates were calculated at a plot level. For metrics such as (normalized) organ-specific biomass (LB, WB and FRB), TB, biomass allocation to individual organs (LMF, SMF and RMF), LAI, and crown shape parameters, analyses were carried out either at the level of species (per plot) or at the plot level. RAI, LAI/RAI and allocation ratios, such as LB/DBH and FRB/DBH, were assessed in a similar manner. Graphs were compiled using the ‘ggplot2’ package (Wickham, 2016). Outliers, defined as observations deviating significantly from the central tendency by falling below the first/third quartile ± (1.5 × interquartile range), were removed prior to analysis. These outliers accounted for 0.5–4.6 % of the data. Unless otherwise stated, the values given are the mean ± s.e.; significance threshold was set at a *P*-value < 0.05; *P*-values < 0.1 are denoted as trends.

## RESULTS

### Basal area and height growth, and survival rates during stand establishment

**Fig. 2.**
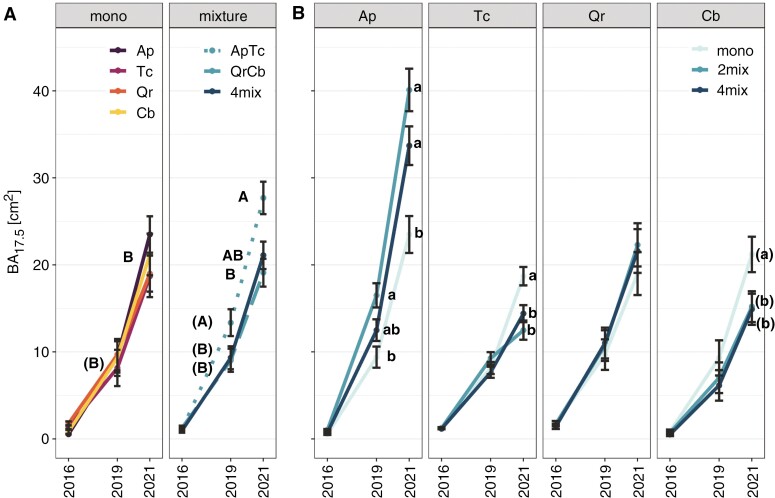
Stem basal area (BA_17.5_) of tree individuals per species, diversity level and year; planting took place in 2013. (A) Seven plot types: monocultures (*Acer platanoides*, Ap; *Carpinus betulus*, Cb; *Quercus robur*, Qr; *Tilia cordata*, Tc; ‘mono’), two-species (ApTc, QrCb; ‘2mix’) and four-species (‘4mix’) mixtures. (B) Species-specific BA_17.5_ by diversity level; species and/or diversity level are colour coded. Different lowercase letters indicate significant differences (*P* < 0.05; LMM with Tukey’s *P*-value adjustment; *n* =2355–3279; mean ± s.e.) between diversity levels per species and year; uppercase letters (in A) indicate significant differences between monocultures and mixture plots within a single year. Letters in parentheses denote trends (*P* < 0.1).

The basal area (BA_17.5_) of the four tree species in monoculture increased at a similar rate throughout 2016–2021, but it was strongly affected by the diversity level (Fig. 2A). In 2019, as early as 6 years after planting, *Acer* trees in the mixture with *Tilia* (Ap_2mix_) had a significant, ~1.8 times higher BA_17.5_ compared with Ap_mono_ trees (Fig. 2B); the difference between diversity levels became more pronounced in 2021, resulting in significantly greater Ap_2mix_ and Ap_4mix_ compared with Ap_mono_. In contrast, *Tilia* and *Carpinus* monocultures (Tc_mono_ and Cb_mono_) had ~1.4 times greater BA_17.5_ than in their respective mixtures, and no significant differences in BA_17.5_ of *Quercus* (Qr) were found at any diversity level. Up to 8 years after planting, tree heights of all species, besides *Carpinus*, were unaffected by diversity levels (Supplementary Data Fig. S5A).

Over the monitored period, survival rates did not differ significantly among diversity levels; the average mortality rate per species ranged between 0.2 and1.4 % year^−1^, with a greater mortality of Ap and lowest mortalities of Tc and Qr trees (Supplementary Data Table S2 and Fig. S6).

### Effects of mixtures on biomass

**Fig. 3.**
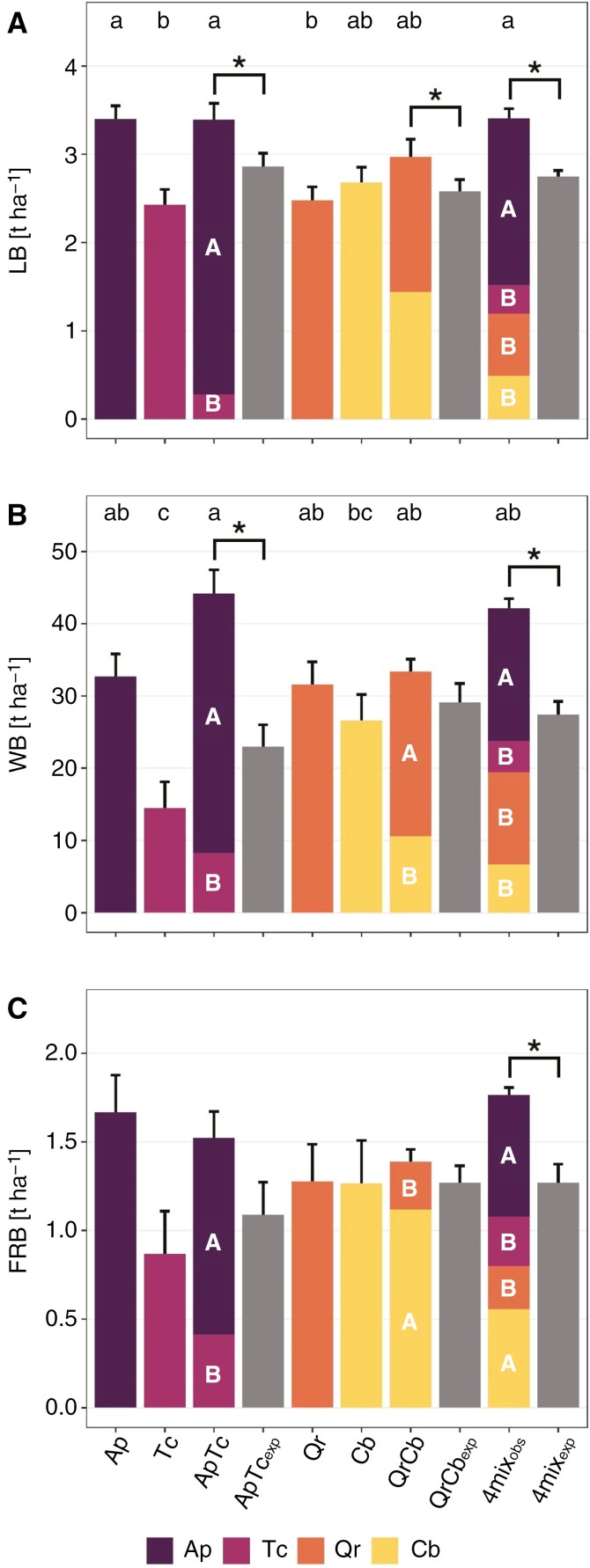
Organ- and tree species-specific biomass of leaves (LB) (A), wood (WB) (B) and fine roots (FRB) (C) per plot type. Colours denote observed species-specific biomass. Expected biomass (‘exp’, grey), i.e. as derived from respective monocultures (for details, see Materials and methods), is given for mixtures. Lowercase letters indicate significant differences between plot types (*P* < 0.05; LMM with Tukey’s *P*-value adjustment; *n* = 25; mean ± s.e.). Uppercase letters indicate significant differences between the contribution of each tree species to the total biomass of the respective mixture type. Asterisks indicate significant differences between observed and expected biomass of mixtures. Plot types: tree species monocultures (*Acer platanoides*, Ap; *Carpinus betulus*, Cb; *Quercus robur*, Qr; *Tilia cordata*, Tc), two-species mixtures (ApTc and QrCb) and the four-species mixture (‘4mix’).

The highest total leaf biomass (LB) was found in the four-species mixture, Ap_mono_ and ApTc 2mix plots (Fig. 3A). The smallest LB was found in the monocultures of Tc and Qr. *Acer* in both mixtures (Ap_2mix_ and Ap_4mix_) produced a ~2 times higher LB than in respective monocultures; *Tilia* had a 2–4 times lower LB when in mixture (Fig. 3B). At plot level, both the two- and the four-species mixtures had significantly more leaves (~20 %) than monocultures (Fig. 3B). Supplementary Data Fig. S7 shows all observed versus expected biomass values per organ and plot type.

The ApTc and 4mix plots had the highest biomass in stems and branches (WB; Fig. 3C). Ap_mono_ had the highest WB of the monocultures, followed by slightly lower values of Qr_mono_ and Cb_mono_; Tc_mono_ had a significantly lower WB compared with other species and mixtures. Ap contributed 82 % of the WB of ApTc, and Qr held a two-thirds share of WB in QrCb. Likewise, Tc and Cb made the lowest contribution to total WB in the four-species mixture. Admixing resulted in a significant overyielding of Ap_2mix_ and Ap_4mix_, with ~2.3 times higher WB in the mixtures than expected from Ap_mono_ (Supplementary Data S7D). Qr showed a significantly higher WB (1.4 times) in QrCb than in Qr_mono_; a similar trend (*P* < 0.1) was found for Qr_4mix_. At the plot level, and considering mortality, we observed significant overyielding in WB of ApTc and the four-species mixtures (1.9 and 1.5 times greater, respectively; Supplementary Data Fig. S7D).

The fine root biomass (FRB) to a depth of 40 cm was greatest in the 4mix and Ap_mono_ plots; Tc_mono_ had the lowest FRB (Fig. 3C); yet differences were statistically not significant. In ApTc, Ap contributed 73 % of the fine roots, significantly more than Tc (Fig. 3C), which is consistent with the pattern observed for leaf and stem biomass (Fig. 3A, B). However, in contrast to the above-ground pattern, Cb in 2mix (QrCb) plots accounted for a significant larger proportion of FRB (81 %) than Qr. In the four-species mixture, both Cb and Ap had the greatest proportions of fine root biomass. Our data indicate a significant overyielding of fine roots in Cb_2mix_ and Cb_4mix_, with FRB 1.8 times higher than expected from Cb_mono_ (Supplementary Data Fig. S7F). Simultaneously, Qr_2mix_ had only 42 % of the FRB as expected from monocultures. The total FRB observed in 4mix was significantly higher, by 40 %, than would be expected from the four monocultures (Supplementary Data S7F). Coarse root biomass (CRB) ranged from 5.13 ± 0.9 t ha^−1^ in Qr_mono_ to 2.06 ± 1.1 t ha^−1^ in Cb_mono_ (Supplementary Data Fig. S8A). No significant differences in CRB were found between plot types or diversity levels (Supplementary Data Fig. S8).

In 2021, the greatest total plant biomass (TB) was found in the ApTc and the four-species mixture, with total biomass ~44 and 12 % higher, respectively, than expected from the respective monocultures. The Tc_mono_ plots had the significantly lowest TB (Supplementary Data Fig. S3). Species-specific biomass components per plot type are given in Supplementary Data Table S2.

### Allocation of biomass and absorbing surfaces, and space utilization

#### Mass fractions.

**Fig. 4.**
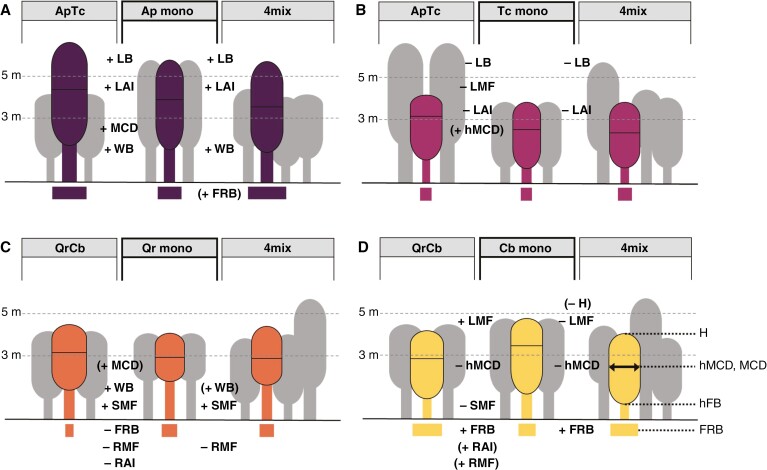
Species-specific biomass allocation, crown shapes and absorbing surfaces. Trees in monocultures (middle) compared with two-species mixtures (left) and four-species mixtures (‘4mix’, right) for: (A) *Acer platanoides* (Ap); (B) *Tilia cordata* (Tc); (C) *Quercus robur* (Qr); and (D) *Carpinus betulus* (Cb); grey shapes illustrate competing species above ground. Parameters from tree top to roots: H = tree height, LB = leaf biomass (in tons per hectare), LMF = leaf mass fraction, LAI = leaf area index, (h)MCD = (height of) maximum crown diameter, WB = wood biomass (in tons per hectare), SMF = stem and branch mass fraction, hFB = height of first branch, FRB = fine root biomass (in tons per hectare), RMF = fine root mass fraction and RAI = fine root area index. The parameters H, hMCD, MCD, hFB and FRB are represented to scale. A ‘+’ denotes a significant increase in the respective parameter, a ‘−’ denotes a significant decrease compared with monocultures (*P* < 0.05; LMM with Tukey’s *P*-value adjustment; mean ± s.e.); parameters in parentheses show trends (*P* < 0.1). FRB, RMF and RAI have been tested across both soil horizons.

**Fig. 5.**
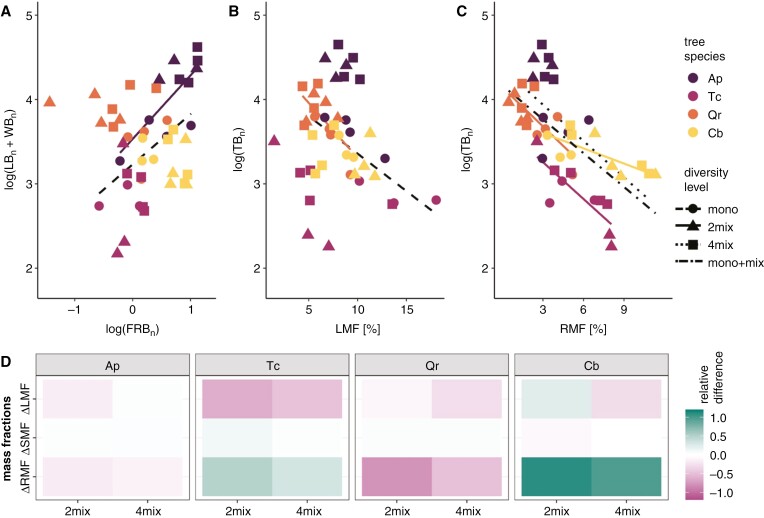
(A) Relationships between log-transformed above-ground biomass [normalized leaf biomass (LB_n_) + normalized wood biomass (WB_n_)] and log-transformed normalized fine root biomass (FRB_n_) per tree species (Ap = *Acer platanoides*, Cb = *Carpinus betulus*, Qr = *Quercus robur* and Tc = *Tilia cordata*) and diversity levels, i.e. monoculture (circle), two-species (‘2mix’; triangle) and four-species mixtures (‘4mix’; square); ‘mono+mix’ represents all data points. (B, C) Relationships between log-transformed normalized total biomass (TB_n_) and leaf mass fraction (LMF) (B) or root mass fraction (RMF) (C) per tree species and diversity level. Lines in A–C indicate significant (*P* < 0.05) linear relationships; coefficients of determination for A: *R*^2^_Ap_ = 0.53 and *R*^2^_mono_ = 0.42; for B: *R*^2^_Qr_ = 0.43 and *R*^2^_mono_ = 0.69; and for C: *R*^2^_Tc_ = 0.67, *R*^2^_Qr_ = 0.51, *R*^2^_Cb_ = 0.58, *R*^2^_4mix_ = 0.48 and *R*^2^_mono+mix_ = 0.41. (D) Changes in leaf (ΔLMF), stem (ΔSMF) and fine root mass fraction (ΔRMF) per tree species and diversity level compared with the respective monocultures. Within the colour gradient of the heatmap, green indicates a positive change, magenta a negative change and white no change.

Biomass allocation showed species-specific patterns (Figs 4 and 5). In two-species mixtures, *Tilia* had a significant lower leaf mass fraction (LMF) compared with Tc_mono_ (Fig. 4B; Supplementary Data Table S3). *Quercus* in two- and four-species mixtures had a significantly greater stem and branch mass fraction (SMF) relative to individuals in monocultures, a pattern that was contrasted in the fine root biomass fraction (RMF), with Qr_mono_ exhibiting a higher RMF than in both mixtures (Fig. 4C). Cb showed significant differences in biomass allocation to leaves, wood and fine roots at different diversity levels (Fig. 4D). The LMF was highest in Cb_2mix_, followed by Cb_mono_; Cb_4mix_ showed a significant decrease in LMF compared with Cb_mono_. Conversely, the SMF was lowest in Cb_2mix_ in comparison to both Cb monoculture and Cb_4mix_. Thus, Cb, when mixed with Qr, showed a significant decrease in SMF and a concomitant increase in RMF. These patterns indicate that both *Quercus* and *Carpinus* exhibit a high degree of phenotypic plasticity in their biomass allocation when grown as mixtures compared with monocultures. In contrast, Ap showed no differences in mass fractions across diversity levels (Fig. 4A). We studied the systematic relationship between above-ground biomass (LB_n_ + WB_n_) and FRB_n_ and found, on a logarithmic scale, a significant positive linear relationship for *Acer* (*R*^2^_Ap_ = 0.53; *P* < 0.05) and when considering the monocultures of all species together (*R*^2^_mono_ = 0.42; *P* < 0.05; Fig. 5A). Allometric relationships between logarithmic TB_n_ and LMF were significant only for monocultures across all species (*R*^2^_mono_ = 0.69, *P* < 0.05) and *Quercus* (*R*^2^ = 0.43, *P* < 0.05; Fig. 5B). Negative allometric relationships between log-scale TB_n_ and RMF are suggestive that plots with bigger Tc, Qr and Cb trees hold a significantly lower RMF (*R*^2^ = 0.51–0.67; *P* < 0.05; Fig. 5C). The slopes of these linear relationships differed markedly between *Quercus* (~−6.2 times), *Carpinus* (−1.9 times) and *Tilia* (−2.8 times). No significant relationships between TB_n_ and RMF were found for *Acer* and across monocultures. The changes in mass fractions translated into species-specific differences in mixtures when compared with their respective monocultures (Fig. 5D). Although the relative differences in SMF remained relatively constant, with ΔSMF varying between −0.08 and 0.11, a general decrease in LMF was observed in mixtures, with a maximum ΔLMF of −0.62 (in Tc_2mix_), except for *Carpinus* in two-species mixtures, where an increase in ΔLMF of 0.23 was observed. In terms of RMF, both Tc and Cb showed an increased relative allocation to fine root biomass in mixtures (to ΔRMF maxima of 0.49 and 1.1, respectively). In contrast, the larger Qr in both mixtures showed a reduced ΔRMF up to −0.8 when compared with Qr monocultures. Controlling for tree size by including TB or TB_n_ as a covariate in our models yielded similar results (data not shown).

#### Absorbing surface areas and their relationship to the stem diameter.

**Fig. 6.**
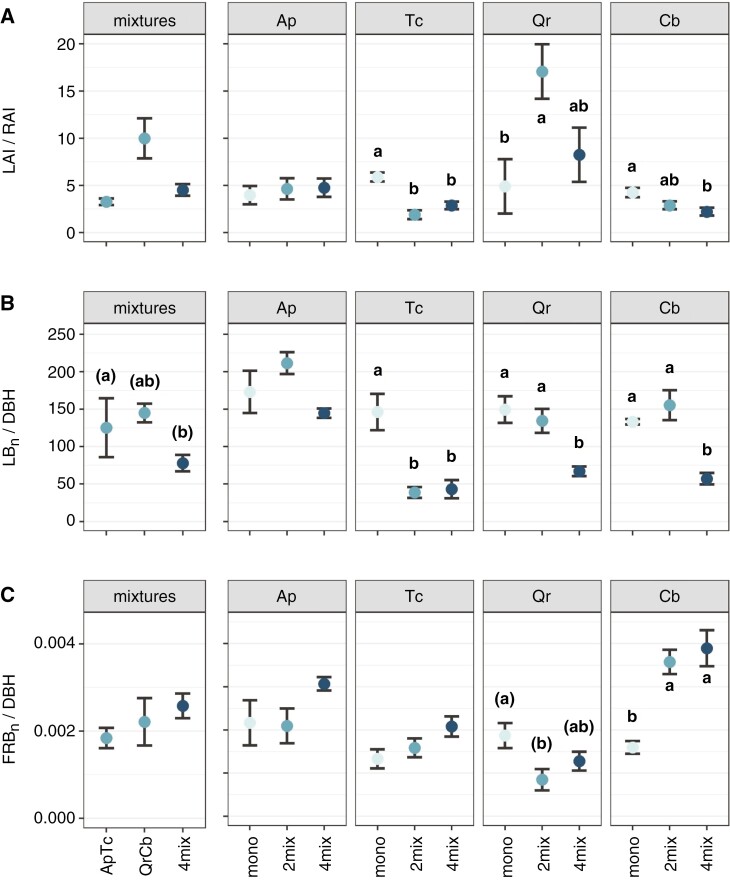
Ratios of absorbing surfaces above to below ground or biomass towards the stem diameter. (A–C) Leaf area to root area (LAI/RAI) ratio (A), normalized leaf biomass (LB_n_) to diameter at breast height (DBH) (B) and normalized fine root biomass (FRB_n_) to DBH (C) per tree species and plot type, respectively, and diversity level. Species are *Acer platanoides* (Ap), *Carpinus betulus* (Cb) *Quercus robur* (Qr) and *Tilia cordata* (Tc) in monoculture, two-species (‘2mix’) and four-species mixture (‘4mix’). Letters indicate significant differences (*P* < 0.05; LMM with Tukey’s *P*-value adjustment; *n* = 10–12; mean ± s.e.); letters in parentheses denote trends (*P* < 0.1).

At the plot level, both the highest total leaf area index (LAI) and root area index (RAI), of 6.1 ± 0.3 and 1.6 ± 0.3 m^2^ m^−2^, respectively, were found in four-species mixtures (Supplementary Data Fig. S9). These differences were largely based on species-level changes in leaf and fine root biomass, because we found no effects of diversity level on either SLA or SRA, except for SLA in Tc_2mix_ (Supplementary Data Fig. S10). Species- and organ-specific differences resulted in diversity level-specific changes in the ratio of above-ground to below-ground absorbing surfaces (Fig. 6A). A significant decrease of LAI/RAI ratios with stem size (DBH) was found across monocultures (*R*^2^_mono_ = 0.47, *P* < 0.05) but not for mixtures (Supplementary Data Fig. S4A). Both Tc and Cb had significantly lower LAI/RAI ratios in mixtures; LAI/RAI was, for example, 68 % lower for Tc_2mix_ than for Tc_mono_ (Fig. 6A) despite being only ~14 % different in normalized above-ground biomass. In contrast, the taller Qr_2mix_ trees had a 3.3 times greater LAI/RAI ratio than Qr_mono_. Although no significant changes in the leaf to root area ratios were found for *Acer*, which had the highest LAI of all monocultures, both Ap_2mix_ and Ap_4mix_ had ~2 times significantly greater LAI_n_ values than expected from monoculture (Supplementary Data Fig. S9A). Although the LAI_n_ of Tc_mono_ was already one of the lowest (4.5 m m^−2^), it was significantly reduced to 1.1 m^2^ m^−2^ in Tc_2mix_. Given that the LAI_n_ of neither Qr nor Cb changed significantly in mixture, altered LAI/RAI was driven by changes in fine root surface area. In addition, LAI/RAI ratios exhibited a significantly positive relationship to DBH in Cb (*R*^2^_Cb_ = 0.68, *P* < 0.05; Supplementary Data Fig. S4A), but not in the other species. Both Qr_2mix_ and Cb_2mix_ had significantly lesser (−65 %) or higher (+57 %) RAI_n_, respectively, compared with monocultures. Ap_mono_ had the highest RAI_n_ to 40 cm soil depth, whereas Tc_mono_ had the lowest (Supplementary Data Fig. S9B).

The leaf biomass relative to stem diameter (LB/DBH) ratio was low for all four species in high diversity (4mix) stands compared with monocultures (Fig. 6B). The LB_n_ increased significantly with DBH across 4mix plots and across all diversity levels (*R*^2^ = 0.59–0.84, *P* < 0.05; Supplementary Data Fig. S4B) and at a species level for *Carpinus* (*R*^2^_Cb_ = 0.38, *P* < 0.05). No significant relationships between LB_n_ and DBH were found across monocultures and for Ap, Tc and Qr (Supplementary Data Fig. S4B). Interestingly, normalized fine root biomass relative to the stem diameter (FRB_n_/DBH) showed a diverging pattern from LB_n_/DBH for Tc, Qr and Cb. *Carpinus* trees in mixtures had a significantly greater FRB_n_/DBH ratio compared with the monoculture (Fig. 6C), and FRB_n_ showed a significant negative relationship to DBH in *Carpinus* only (*R*^2^_Cb_ = 0.49, *P* < 0.05). Across species, FRB_n_ increased (significantly) with DBH (*R*^2^ = 0.12–0.26; Supplementary Data Fig. S4C).

#### Crown shape and root system segregation.

Crown shape varied with species, and partially with diversity level (Fig. 4; Supplementary Data Table S2). For example, the maximum crown diameter (MCD) of *Acer* in the two-species mixture was significantly larger than in the corresponding monocultures and 4mix (Fig. 4; Supplementary Data Fig. S5D). A similar trend (*P* < 0.1) was found for *Quercus*. *Carpinus* showed a significant plasticity in the height of the maximum crown diameter (hMCD; Supplementary Data Fig. S5B), with a significantly lower hMCD in both mixtures compared with Cb monocultures. The height of the first living branch (hFB), i.e. the lower end of the canopy, was unaffected by the diversity level (Supplementary Data Fig. S5B).

**Fig. 7.**
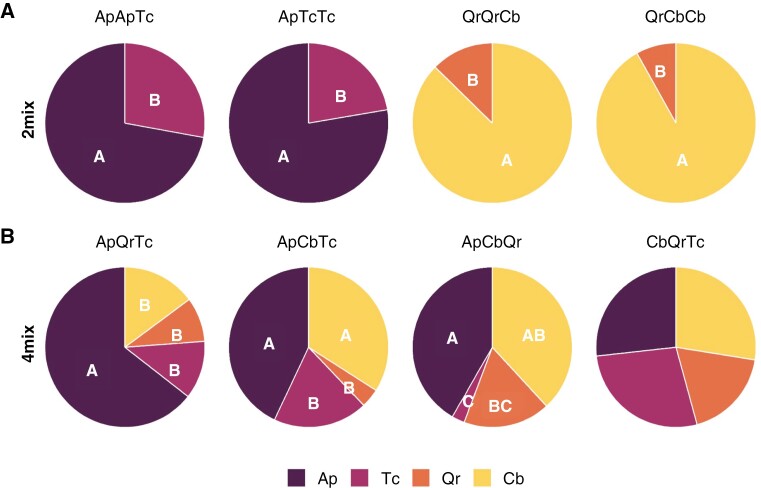
Relative biomass proportions by tree species to the total fine root biomass per tree triplet centres in: (A) two-species mixtures (2mix); and (B) four-species mixtures (4mix) for the species *Acer platanoides* (Ap), *Carpinus betulus* (Cb), *Quercus robur* (Qr) and *Tilia cordata* (Tc). The surrounding trees per sample point in the centre of tree triplets are given; e.g. ‘ApApTc’ denotes a location surrounded by two Ap and one Tc trees. Proportions are averages of 0–20 and 20–40 cm soil depth; no significant differences were found between soil depths (data not shown). Different letters indicate significant differences (*P* < 0.05; LMM with Tukey’s *P*-value adjustment; *n* = 6–12; mean ± s.e.).

Looking at use of below-ground space, total FRB was on average ~40 % greater in the upper mineral soil layer (0–20 cm) than in the 20–40 cm layer (Supplementary Data Fig. S11A). Consequently, the RAI_n_ was ~1.5 times greater in the topsoil than in the subsoil (Supplementary Data Fig. S12). This pattern remained similar across species and diversity levels; no significant changes in vertical rooting pattern between soil horizons were found (Supplementary Data Fig. S11). In contrast, we found large differences in the horizontal distribution of fine roots depending on the triplet sampled. For example, the contribution of species to the FRB in two-species triplets, i.e. a soil core surrounded by two species in 2mix (Fig. 1D), clearly showed that roots of either *Acer* or *Carpinus* significantly dominated over *Tilia* or *Quercus*, respectively. Ap and Cb composed 72–78 and 87–92 % of the FRB within the 2mix core, respectively, regardless of the number of conspecific trees (i.e. one or two in the triplet; Fig. 7A). *Acer* roots provided a large fraction of total FRB even in the absence of Ap from the surrounding triplet of trees (CbQrTc; Fig. 7B); Ap was found in ~22 % of these 4mix soil cores (Supplementary Data Table S3). When Ap and Cb were both part of the 4mix species triplet (i.e. ApCbTc and ApCbQr), they co-dominated the rooting space by exhibiting similar proportions of fine root biomass (34–43 %). In the absence of Cb (ApQrTc), however, *Acer* roots dominated the 4mix rooting space, contributing 64% of FRB (Fig. 7B). Likewise, Ap and Cb accounted for a greater-than-expected share of the fine root biomass within 2mix soil cores in 88 and 96 % of the cases, respectively (Supplementary Data Table S4). In four-species mixtures, 76 % of all cores held fine roots from three or more species, irrespective of the triplet (data not shown). Qr roots were present in ~12 % of the cores within four-species mixtures while not growing in the immediate vicinity (i.e. ApCbTc), indicating proficient soil exploration, albeit with generally modest biomass contributions.

## DISCUSSION

Our study contributes to the growing body of research on biodiversity–productivity relationships in planted mixed-species forests, using potential target species suitable for diversifying Central European forests under progressive climate change (Leuschner *et al.*, 2024). We show that species identities are key drivers of yield effects during early stand development and that diversity levels influence not only above-ground yield but also fine root biomass. Finally, we highlight the acclimatization of key traits of individual species within mixtures, both in terms of allocation patterns and positioning of absorbing organs or their surprising absence; in sum, underlying the observed community-level yield effects.

### Yield effects during early stand development advance rapidly

Many studies have shown that mixed plantations can outperform single-species stands and that such ‘overyielding’ effects continue to increase with time (Pretzsch and Schütze, 2009; Thurm and Pretzsch, 2016; Tatsumi, 2020; Dietrich *et al.*, 2023). To date, overyielding has been reported mainly in mature forests (Thurm and Pretzsch, 2016; Shu *et al.*, 2018) or in planted mixtures of deciduous and evergreen species (Urgoiti *et al.*, 2022, 2023*b*), while studies on the temporal development of yield effects in planted mixed deciduous forests are scarce. Not unexpectedly, our results suggest that canopy closure and incipient competition are necessary to cause significant yield effects in most of the deciduous species in mixture, i.e. 8 years after planting. Similar, earlier studies (although conducted in mixtures of both deciduous and conifer species), such as those by van de Peer *et al.* (2017*a*) and Urgoiti *et al.* (2023*b*), also reported overyielding effects within 6–9 years after establishment. As hypothesized, the ‘acquisitive’ *Acer platanoides* (Ap) grew significantly better when in admixture with heterospecific neighbours starting from the onset of canopy closure (i.e. 6 years after planting), and this effect strengthened subsequently. Urgoiti *et al.* (2023*b*) reported that deciduous species with acquisitive traits performed, on average, better in mixtures relative to monocultures than evergreen species with conservative traits. In contrast, our study using deciduous broadleaved species along an acquisitive–conservative gradient of life-history strategies cannot confirm this. Here, surprisingly and contrary to our first hypothesis, the intermediate acquisitive species *Tilia cordata* (Tc) and *Carpinus betulus* (Cb) both grew worse in mixtures than in monocultures, whereas the ‘conservative’ *Quercus robur* (Qr) showed no negative response in basal area and height growth with increasing diversity (but significantly overyielding wood biomass 8 years after planting; see below). The successional niche hypothesis proposes that early in the succession, competitive ability is characterized by high productivity, whereas later in the succession it is characterized by the ability to persist under low resource availability (Goldberg, 1996; Pacala and Rees, 1998; Reich, 2014). Thus, although the basal area growth in monocultures was very similar among the studied species, it was probably the superior height growth of young *Acer* trees that allowed them to pre-empt light by positioning a large part of their crowns above those of competitors (see also discussion below). These differences in growth performance in mixtures did not (yet) translate to differences in survival rates. This aligns with previous research in younger mixed plantations, where a neutral or stabilizing rather than a positive influence of tree richness on survival rates has been found (Liang *et al.*, 2007; Grossman *et al.*, 2018; Blondeel *et al.*, 2024). Searle *et al.* (2022) and other studies have linked higher tree mortality rates to increased stem densities in mixed plantations. However, the planting density in the B-Tree experiment, ~10 500 trees ha^−1^, is homogeneous across diversity levels and moderate compared with other biodiversity–ecosystem functioning studies featured in TreeDivNet (Verheyen *et al.*, 2016). In summary, our findings suggest that early yield benefits in planted mixed forests, particularly those involving early successional, acquisitive species, such as *Acer platanoides*, can be detected and that the yield effects originate from growth modulation rather than a reduced mortality.

### Mixed-species stands possess yield effects across organs

In our study, we observed overyielding (the phenomenon whereby mixed-species stands exhibit greater community productivity compared with their respective monocultures) across biomass compartments, i.e. including wood, leaf and fine root biomass. This extends previous findings of positive biodiversity–productivity relationships (Pretzsch and Schütze, 2009; Williams *et al*., 2017; Lu *et al.*, 2018; but see Ahmed *et al.*, 2019) by demonstrating species-differentiated yielding effects across plant organs.

Our results partly support our second hypothesis, showing that the highest diversity level, i.e. the four-species mixture, persistently exhibited overyielding in all compartments. Although yield in the two-species mixtures generally followed the same pattern across organs as in the four-species mixtures, overyielding was less pronounced below ground, highlighting a nuanced response to neighbour identity. *Acer platanoides* contributed particularly to above-ground overyielding in mixtures, but overyielding effects were also visible below ground (only partly significant owing to the very high variability). This dominance enabled the acquisitive *Acer* to outcompete species such as *Tilia*, aligning with earlier findings from van de Peer *et al.* (2017*b*). In fact, the interspecific competition intensity towards *Tilia* in mixtures was so pronounced that *Tilia* was the only species in the portfolio with significantly less leaf biomass compared with the monoculture. Similar results were reported for mixtures of pine and birch, whereby birch reduced in growth, whereas pine benefitted from the alleviation of the intraspecific competitive inhibition of crown growth (Martin-Blangy *et al.*, 2023). Although the QrCb mixture did not show significant overyielding at the community level, *Quercus* stem wood alone did, hinting at modified competition intensity by the heterospecific neighbours (Fichtner *et al.*, 2017). Supporting this, Ray *et al.* (2023) emphasize how biodiversity increases productivity in mixed forests through the interplay of the light-capture strategies of the shade-tolerant and light-demanding species. Such differences in strategic adaptations for light acquisition, as noted by Ray *et al.* (2023), are key drivers of growth patterns. In contrast to the light-demanding *Quercus*, *Acer* showed yield effects above ground at both leaf and stem levels. In accordance, earlier studies have shown that species with a higher SLA, such as *Acer*, tend to exhibit greater phenotypic plasticity in varying environmental conditions (Stotz *et al.*, 2022), potentially including those imposed by heterospecific neighbours. However, studies on leaf biomass yields in mixed forest stands remain scarce (see e.g. Williams *et al.*, 2017; Steinparzer *et al.*, 2022), making it difficult to predict reaction norms as driven by species-specific strategies for light interception and light-use efficiency (Williams *et al.*, 2021), and the complementarity of light-capture strategies among coexisting species. For example, given that *Tilia cordata* is considered to be (very) shade tolerant (Pigott, 1991) and the mortality rates remained low, it cannot yet be concluded that the reduced leaf biomass inevitably indicates the future competitive exclusion of *Tilia* from the mixtures.

Although tree species diversity effects on above-ground biomass are increasingly investigated, below-ground responses, such as changes in FRB, are still considerably less explored. This is surprising because fine roots have a decisive effect on both plant resource supply and ecosystem functioning (Freschet *et al.*, 2021). The present results on mixture effects on FRB are inconsistent. For example, Lei *et al.* (2012*a*), Valverde-Barrantes *et al.* (2015), Shu *et al.* (2018) and Schuster *et al.* (2023) reported positive correlations between FRB and species diversity, indicating overyielding in planted forests. In contrast, research in mature stands often does not find significant below-ground overyielding (Meinen *et al.*, 2009; Jacob *et al*., 2013; Fruleux *et al.*, 2018; Lwila *et al.*, 2021). Domisch *et al*. (2015), studying both standing biomass and root turnover in temperate and boreal plantations, attributed this to rather uniform root traits of admixed species and high nutrient availability in certain forest types. Although derived on a rather nutrient-rich former grassland site, our findings reveal clear overyielding effects in the four-species mixture, with a distinct species-specific pattern for FRB. *Carpinus* and partly also *Acer* contributed most to the community-level fine root biomass, whereas *Quercus* trees with a larger WB possessed significantly fewer fine roots in the two-species mixture (see also discussion below). Similar to earlier findings (Rewald and Leuschner, 2009*a*), the effect of heterospecific neighbours on the FRB of *Tilia* was limited. Interestingly, however, the mixture effect on FRB of two of the three other species is notably distinct from the yielding patterns observed above ground. At the plot level, only monocultures (across species) and *Acer* (across diversity levels) possessed a significantly positive relationship between log-scale FRB_n_ and above-ground biomass. Although admixed *Acer* increased above- and below-ground biomass in parallel (also shown by a rather constant root mass fraction), particularly *Quercus* and *Carpinus* showed strong and contrasting yield effects across organs (see also discussion below). The (size-related) biomass allocation patterns of specific species are discussed in greater detail below. However, our study reveals a clear positive biodiversity–productivity relationship across biomass compartments in a young, planted forest encompassing a wide trait spectrum, from acquisitive *Acer* to conservative *Quercus*. This supports the concept that trait diversity enhances the stand-level productivity of mixed forest ecosystems (Bongers *et al.*, 2021; Zheng *et al.*, 2021).

### Competition leads to size-related patterns in above- and below-ground biomass allocation, but species-specific shifts in space utilization and inter-organ scaling

Competition for resources such as light, water and nutrients can manifest itself in various ways, either symmetrically, whereby species acquire resources proportionally to the biomass allocated, or asymmetrically, with one species dominating resource capture, for example through shading. Root systems compete by their ability to explore soil and exploit resource-rich patches, and this competition is considered to be more size symmetric than competition for light (Rewald and Leuschner, 2009*b*; Rasmussen *et al.*, 2019; Lak *et al.*, 2020). Although we must not neglect the importance of other factors, such as carbon storage, for long-term persistence, particularly under shade (e.g. Petrovska *et al.*, 2021), and/or species-specific efficiencies in building and maintaining tissue (e.g. Niinemets, 1998; Rewald *et al.*, 2014), carbon allocation above and below ground remains a key factor governing resource competition. We had thus hypothesized that biomass allocation into leaves and fine roots is highly plastic under competition, as a function of both the intensity of competition and the ability of species to acclimatize, although we were aware of potential biomechanical and/or hydraulic constraints (e.g. Berry *et al.*, 2024). However, our results support our third hypothesis only in part, as allocation responses to neighbourhood diversity scale to tree size. Interestingly, however, two contrasting patterns emerged. First, the above-ground biomass of acquisitive *Acer* was significantly related to fine root biomass irrespective of diversity levels, resulting in rather stable root mass fractions of ~3–4 % in *Acer*. Second, in contrast, the more conservative species (Cb, Tc and particularly Qr) significantly reduced their RMF with increasing total plant biomass, in accordance with earlier results for monocultures (Poorter *et al.*, 2012 and references within). Biomass allocation in the more conservative species was thus less plastic towards interspecific competition than expected, indicating trade-offs between above- and below-ground investment. We can only speculate that a rather non-limiting supply of photosynthetic assimilates (Prescott *et al.*, 2020), potential lower resource costs (C and N) per unit leaf or root area (for leaves: Niinemets, 1998) and/or the lower C costs for symbiotic fungi allowed the arbuscular mycorrhizal *Acer* to maintain its RMF. The respiration rates per biomass are, however, rather similar in *Acer* and *Tilia* fine roots, with higher/lower respiration rates in *Carpinus* and *Quercus*, respectively (Rewald *et al.*, 2014).

Although growth limits set by resources other than C are reasonable (see e.g. Prescott *et al.*, 2020), B-Tree is a rather nutrient-rich former grassland site (topsoil C/N ratio 19.7 ± 0.7, pH 8.28 ± 0.01). It has been suggested that admixed trees on fertile soil prioritize investments into leaves (Freschet *et al.*, 2015). However, the general slight decrease in ΔLMF seems to imply a reduced interspecific competition intensity for light. Although larger trees (across species and diversity levels) generally had more leaves, as expected, the changes in the ratio between leaf biomass and stem diameter emphasize that neighbour identities significantly affect intra-tree scaling. For example, LB_n_/DBH was significantly smaller in the more conservative species growing in the four-species mixture compared with the respective monocultures. Here, Cb and Tc differed from Qr, because the two rather ‘intermediate’ conservative and shade-tolerant species also had drastically lower DBHs in the four-species mixture, whereas the light-demanding Qr moderately reduced LB_n_ while maintaining DBH similar to monocultures. The lower transpiring leaf mass relative to the water-conducting stem cross-section most probably has important functional consequences for water transport and hydraulic safety (Lübbe *et al.*, 2017), making further investigation of the effects of tree mixtures on water use and drought tolerance imperative. Furthermore, structural acclimatization of canopies, known to be influenced significantly by competition (Pacala and Rees, 1998; Seidel *et al.*, 2011), was highly species and mixture specific. Crown stratification, for example, was evident in both two-species stands, with *Acer* expanding wider crowns above those of *Tilia* without significant height adjustment, whereas *Carpinus* acclimatized to the taller *Quercus* trees by significantly lowering mean crown height. Our data illustrate the subtle but complex changes that occurred at leaf, crown and stem level, supporting the idea that single variables, such as DBH, are increasingly seen as insufficient for predicting crown development (Fu *et al.*, 2013; Qiu *et al.*, 2023; but see Glatthorn, 2021).

Given that the specific root area was unaffected by diversity levels (but species-specific differences remained), the observed changes in leaf to fine root area ratios (LAI/RAI) corresponded to changes in biomass allocation patterns. Lei *et al.* (2012*b*) reported greater below-ground competitive strength in conifers within mixtures, as evidenced by lower LAI/RAI ratios, while root morphology remained unaffected. However, earlier studies have reported varying effects of mixtures on root morphology (e.g. Lwila *et al.*, 2021; Wambsganss *et al.*, 2021*a*, 2021*b*). In our study, *Tilia* and *Carpinus* showed significantly reduced LAI/RAI ratios in mixtures, and conversely, increased root biomass to stem diameter ratios (FRB_n_/DBH). In contrast to the findings of Lwila *et al.* (2021), who reported no significant relationship between FRB and BA in mature stands of *Fagus sylvatica* intermixed with conifers, we found that FRB_n_ showed a significant positive relationship to DBH across species and diversity levels. However, at a species level, only *Carpinus* showed a significant and negative relationship between FRB_n_ and DBH and significantly greater FRB_n_/DBH ratios in mixtures. Without determining their (potentially growth-limiting) resource status, we cannot yet determine whether the pronounced increase in ΔRMF observed in admixed *Carpinus* and, to a lesser extent, in *Tilia* suggests an active shift towards increased below-ground resource acquisition in mixtures or is a ‘passive’ consequence of lower above-ground biomass. We thus recognize the importance of considering the interplay between above- and below-ground biomass and its ontogenetic changes (Poorter and Sack, 2012; Madrigal-González *et al.*, 2016) when interpreting biomass plasticity under competition. However, we speculate that the particularly large changes in ΔRMF of *Tilia* and *Carpinus* and markedly different slopes are related, at least in part, to active species-specific differences in carbon allocation strategies (Imaji and Seiwa, 2010; but see Thompson, 2023). Particularly conservative species with carbon-costly organs (e.g. leaves with a low SLA), such as *Quercus*, might be less flexible in reallocating resources (Stotz *et al.*, 2022), resulting in the negative relationship between total plant biomass and RMF observed for all species except the acquisitive *Acer*. However, given that the range of mass values differs by diversity levels and is based on a single harvest of roots, we cannot explore this more systematically (Poorter and Sack, 2012).

In contrast to several studies reporting root system stratification/niche separation in interspecific mixtures (Büttner and Leuschner, 1994; Valverde-Barrantes *et al*., 2015; but see Rewald and Leuschner, 2009*a*; Wambsganss *et al.*, 2021*a*), our results indicate no significant horizontal or vertical segregation in the root systems of the four deciduous species. Soil samples consistently contained roots from all neighbouring trees in the immediate vicinity and, occasionally (6–22 % of samples), also from species growing further away, illustrating the establishment of an extensive root system overlap in both soil horizons within 8 years. This differs from findings in mixed conifer–deciduous stands, where root overyielding was linked to spatial niche complementarity (Niklaus *et al.*, 2017; van de Peer *et al.*, 2017*b*; Williams *et al.*, 2017). However, after successful soil exploration, resource acquisition by fine roots ultimately depends on species-specific uptake rates, as modulated by mycorrhizal symbiosis (Power and Ashmore, 1996; Usman *et al.*, 2021), in addition to root branching structure (Rewald *et al.*, 2012). Although information on uptake is lacking, the dominance of *Acer* and *Carpinus* fine roots in the mixed-species cores at least underscores their ability to explore and occupy soil (to 40 cm depth) successfully. *Carpinus* showed a particular strong below-ground competitive ability in the two-species mixture, displacing *Quercus* roots and reducing its fine root biomass to 20 % in comparison to monocultures. Likewise, Leuschner *et al.* (2001) and others suggested a marked competitive suppression of oak roots by *Fagus sylvatica*. Despite suggestions that deep-rooted species, such as *Quercus* (Rosengren *et al.*, 2006), might shift root biomass to deeper soil horizons to avoid competition (Büttner and Leuschner, 1994), our data show no significant difference in *Quercus* rooting when exposed to interspecific competitors between the topsoil and the 20–40 cm soil layer. The mechanisms behind the extensive fine root proportions and lateral spread of *Acer* and *Carpinus* remain speculative. *Acer* might benefit from high carbon availability for growing ‘cheap’ fine roots (tending towards the highest specific root area, and AM mycorrhizal). The earlier bud break in *Carpinus* might support earlier root growth to occupy below-ground space (McCormack *et al.*, 2015).

The significant changes in mass fractions and inter-organ proportions highlight the plasticity of the studied species in acclimatizing to interspecific competition. However, further studies on carbon allocation, limiting resources and molecular signalling (Pierik *et al.*, 2013) are needed to clarify whether these changes are an active acclimatization to resource availability, supporting the optimal partitioning theory (Bloom *et al.*, 1985), or are (partly) a passive response to allometric changes and/or resource partitioning trade-offs. Nonetheless, the alterations in biomass allocation and the adjustments in organ scaling illustrate the profound impact that changes in competitors have on space acquisition and functioning. Given that competitive interactions are highly modulated by environmental conditions (Vospernik *et al.*, 2023), further research on mixed stands across site gradients is necessary to identify the processes and traits underlying species-specific competitive abilities above and below ground.

## Conclusion

Our study in a planted mixed forest provides new insights into biodiversity–productivity relationships, demonstrating overyielding of mixtures at levels of leaves, wood and/or fine roots as early as 6 years after establishment. This research, distinct in its focus on mixtures of deciduous broadleaved species and the combination of above- and below-ground traits, underscores that overyielding occurs despite the absence of clear niche segregation, but also that even in nutrient-rich environments, root competition is a component driving mixed forest stand development. Although our findings highlight the intricate and complex interspecies interactions, they emphasize the importance of a comprehensive approach, including the ‘hidden half’, when assessing the productivity of mixed forests. Our study has implications for forest managers and policy-makers, underscoring the importance of selecting heterogeneous species portfolios to establish productive mixed forests.

## SUPPLEMENTARY DATA

Supplementary data are available at *Annals of Botany* online and consist of the following.

Figure S1: Mean daily air temperature in 2 m above ground (red) and monthly precipitation (blue) at the B-Tree experimental site from the year of planting in 2013 to the last forest inventory in 2021. Figure S2: Aerial view of the B-Tree experimental site. Figure S3: Total plant biomass (TB), calculated as the sum of leaf, wood, coarse root, and fine root biomass. Figure S4: Relationships between tree diameter at breast height. Figure S5: Tree height growth and crown parameters. Figure S6: Average, accumulated mortality. Figure S7: Leaf biomass (LB), wood biomass (WB) and fine root biomass (FRB) of different species and diversity levels. Organ- and species-specific biomass of A) leaf, C) wood and E) fine roots per plot type. Figure S8: Coarse root biomass. Figure S9: Leaf area index and root area index. Figure S10: Specific leaf area and specific root area. Figure S11: Fine root biomass (FRB) of different species and diversity levels, separated by mineral soil horizon (0-20 cm and 20-40 cm). Figure S12: Normalized fine root area index (RAIn), separated by mineral soil horizon. Species. Table S1: allometric models for tree species to estimate wood biomass (WB). Table S2: above- and below-ground parameters per diversity level and tree species [*Acer platanoides* (Ap), *Tilia cordata* (Tc), *Quercus robur* (Qr) and *Carpinus betulus* (Cb)] in monocultures (‘mono’), two-species (‘2mix’) and four-species (‘4mix’) mixtures. Table S3: organ-specific mass fractions per diversity level and component species. Table S4: frequency distribution of species-specific fine root contributions to the total fine root biomass per soil core.

mcae150_suppl_Supplementary_Materials
