# Supplementary material for: Early overyielding in a mixed deciduous forest is driven by both above- and below-ground species-specific acclimatization
Source: Ann Bot. 2024 Sep 23;134(6):1077–96. doi: 10.1093/aob/mcae150 (PMC11687630; doi:10.1093/aob/mcae150)
Supplement: mcae150_suppl_Supplementary_Materials [file mcae150_suppl_supplementary_materials.zip › aob-24255-s03.docx]

**Supplementary Information**

*Climate/ Weather*

Precipitation falls on average at 10 days per month, with the majority of events being light (<5 mm d^-1^). The highest air temperatures occur from June to August, with maximum values of 38 °C; winter months are around 0 °C with occasional frosts down to ‑10 °C and a few days of snow cover. The global radiation in 2021 was 1433.95 W m^-2^ (weather station Adcon A733, Department for Crop Sciences, University of Life Sciences and Natural Resources Vienna).

*Soil Properties*

The soil type is described as Chernozem. The substrate is derived from fine alluvial material of the Danube and is typically well aerated due to the high pore volume (between 50-60% PVol)^(^^[[1]](#endnote-2))^. The low stone content (2‑8 Vol-%) decreases slightly with increasing depth^(1)^. Soil organic carbon stock in 0-60 cm, as measured in 2021, is estimated to be 198 tons per hectare^(1)^. Over the whole experimental area, the plant-available water storage capacity of the top 60 cm of the mineral soil varies between 91 and 110 mm in 2021, depending on bulk density and clay content^(1)^. A gravel body, Quaternary gravel deposits from the Danube, starts at a depth of 1.5 m below ground and reaches down to 8 to 10 m^(^^[[2]](#endnote-3))^. In May 2021, the groundwater at the experimental site was located at a depth of c. 3.2 m^(1)^.

1. ^()^ **Ofner** **MM. 2021**. *Bodenphysikalische und -hydrologische Charakterisierung des B-Tree Versuchsstandortes in Tulln*. Bachelor Thesis, Universität für Bodenkultur, Vienna [↑](#endnote-ref-2)
2. ^()^ **Schuch** **M.** **1973**. *Beiträge zur Hydrogeologie des Tullner Feldes*. Verhandlungen der Geologischen Bundesanstalt,Vienna [↑](#endnote-ref-3)
