# Supplementary material for: Early overyielding in a mixed deciduous forest is driven by both above- and below-ground species-specific acclimatization
Source: Ann Bot. 2024 Sep 23;134(6):1077–96. doi: 10.1093/aob/mcae150 (PMC11687630; doi:10.1093/aob/mcae150)
Supplement: mcae150_suppl_Supplementary_Materials [file mcae150_suppl_supplementary_materials.zip › aob-24255-s04.docx]

**Supplementary Figures**


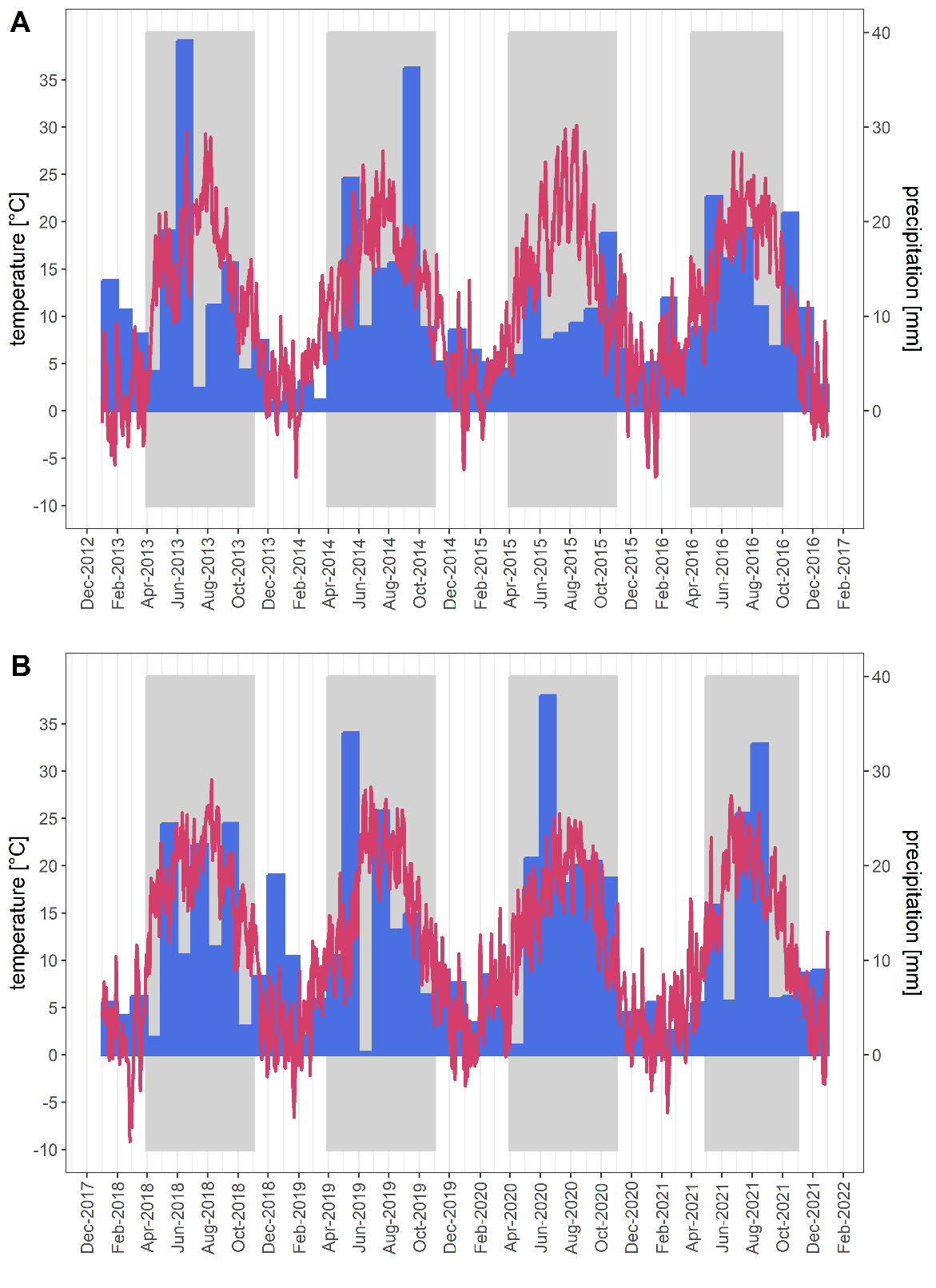


**Fig. S1:** Mean daily air temperature in 2 m above ground (red) and monthly precipitation (blue) at the B-Tree experimental site from the year of planting in 2013 to the last forest inventory in 2021^[[1]](#footnote-2)^. Grey background indicates growing season (length of the period between the first five consecutive days with mean temperature above 5°C and the last of such of the year)^[[2]](#footnote-3)^.


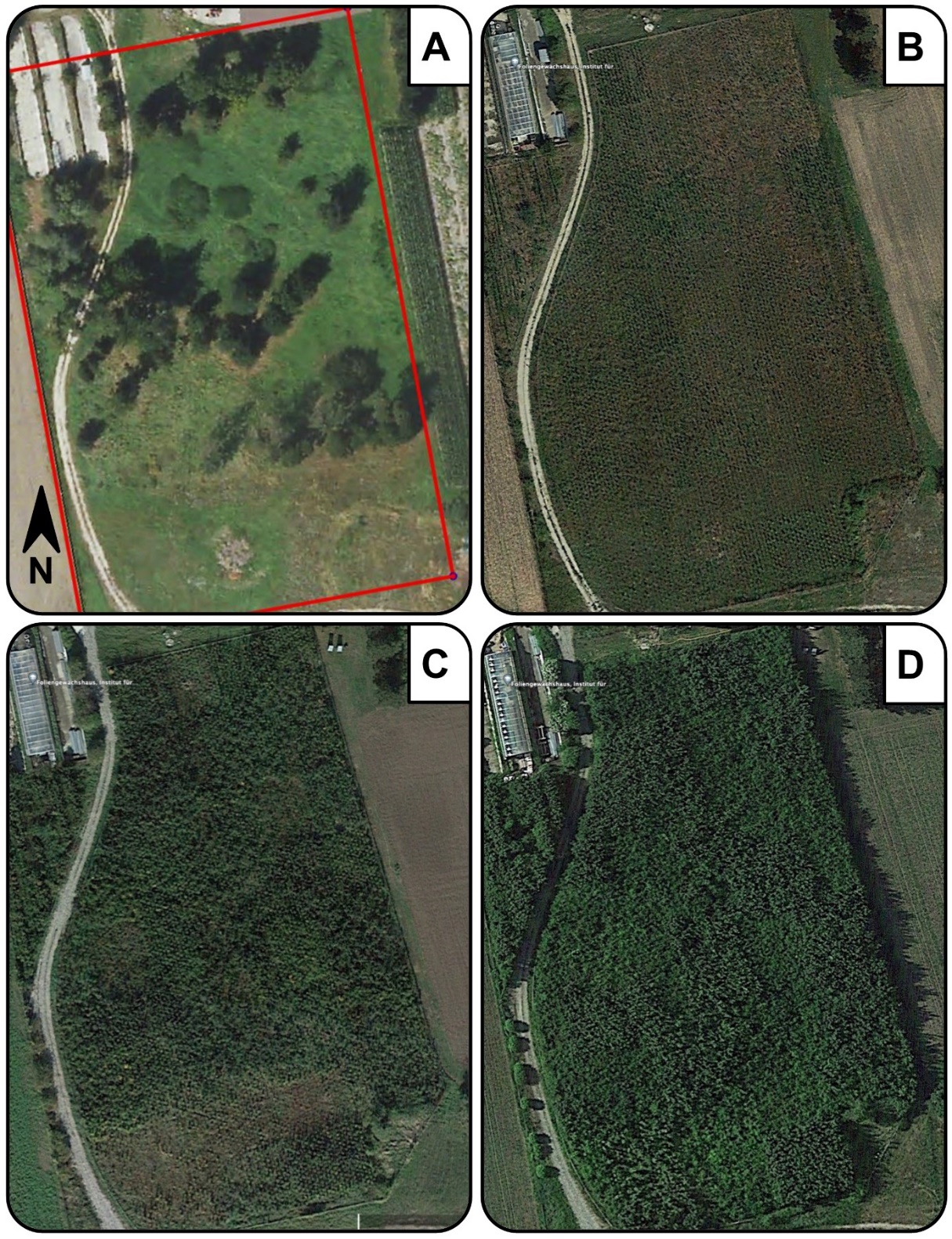


**Fig. S2:** Aerial view of the B-Tree experimental site in **A)** 2011, two years before planting; **B)** 2015, two years after planting; **C)** 2018, five years after planting; and **D)** 2021, eight years after planting. Source: official GIS of the State of Lower Austria.


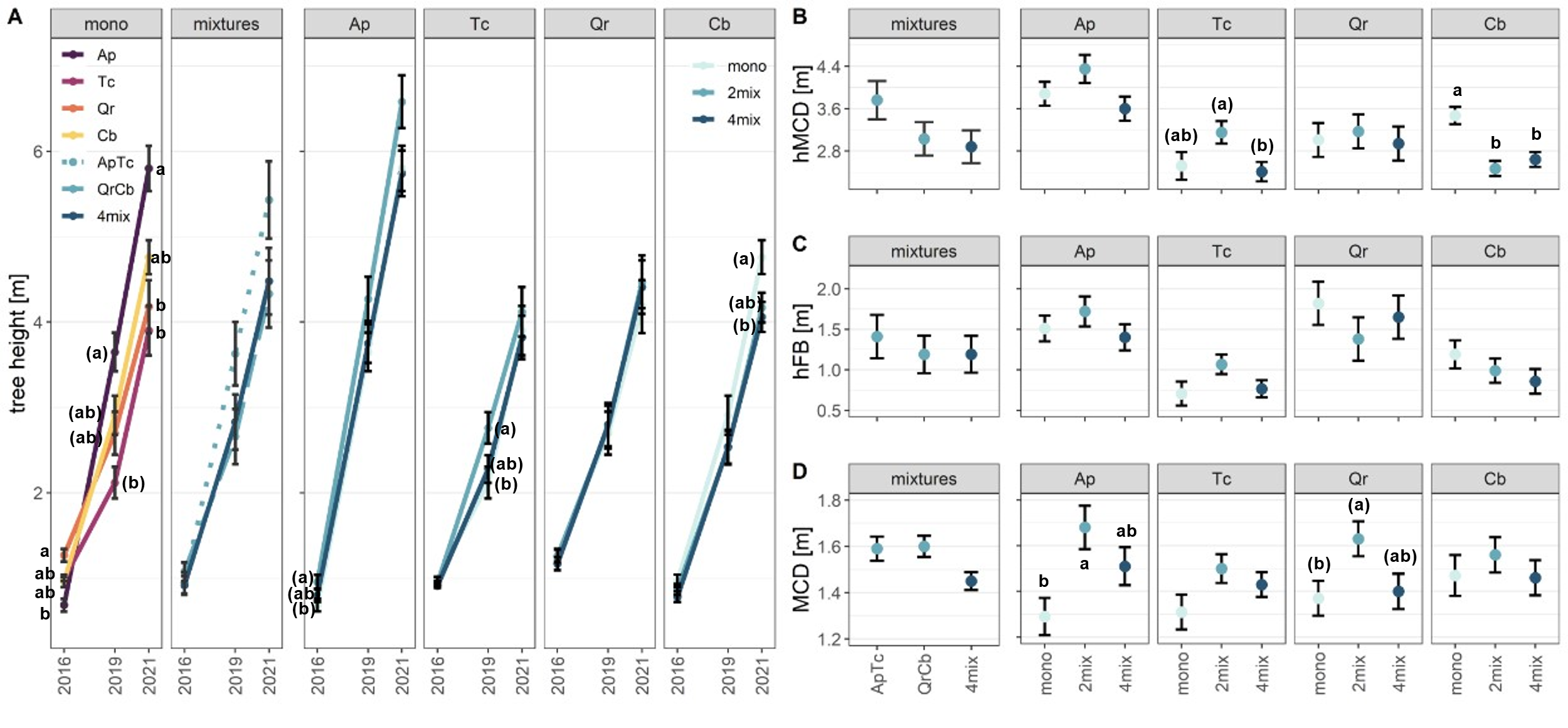


**Fig. S3:** Tree height growth and crown parameters. **A)** average tree height per species, diversity level, and year. Seven plot types: monocultures (*Acer platanoides*, Ap; *Tilia cordata*, Tc; *Quercus robur*, Qr; and *Carpinus betulus*, Cb; ‘mono’), 2-species (ApTc, QrCb; ‘2mix’) and 4-species (‘4mix’) mixtures as well as species-specific heights by diversity level; species and/or diversity level are colour coded. **B)** average height of the maximum crown diameter (hMCD; m), **D)** average height of the first branch (hFB; m), **E)** average maximum crown diameter (MCD; m). Letters indicate significant differences (p <0.05; LMM with Tukey p-value adjustment; n_height_ = 2,343-3,241, n_crown_ _shape_ = 15; mean ± SE), between diversity levels at plot level or per species (and year), letters in parentheses denote trends (p <0.1).


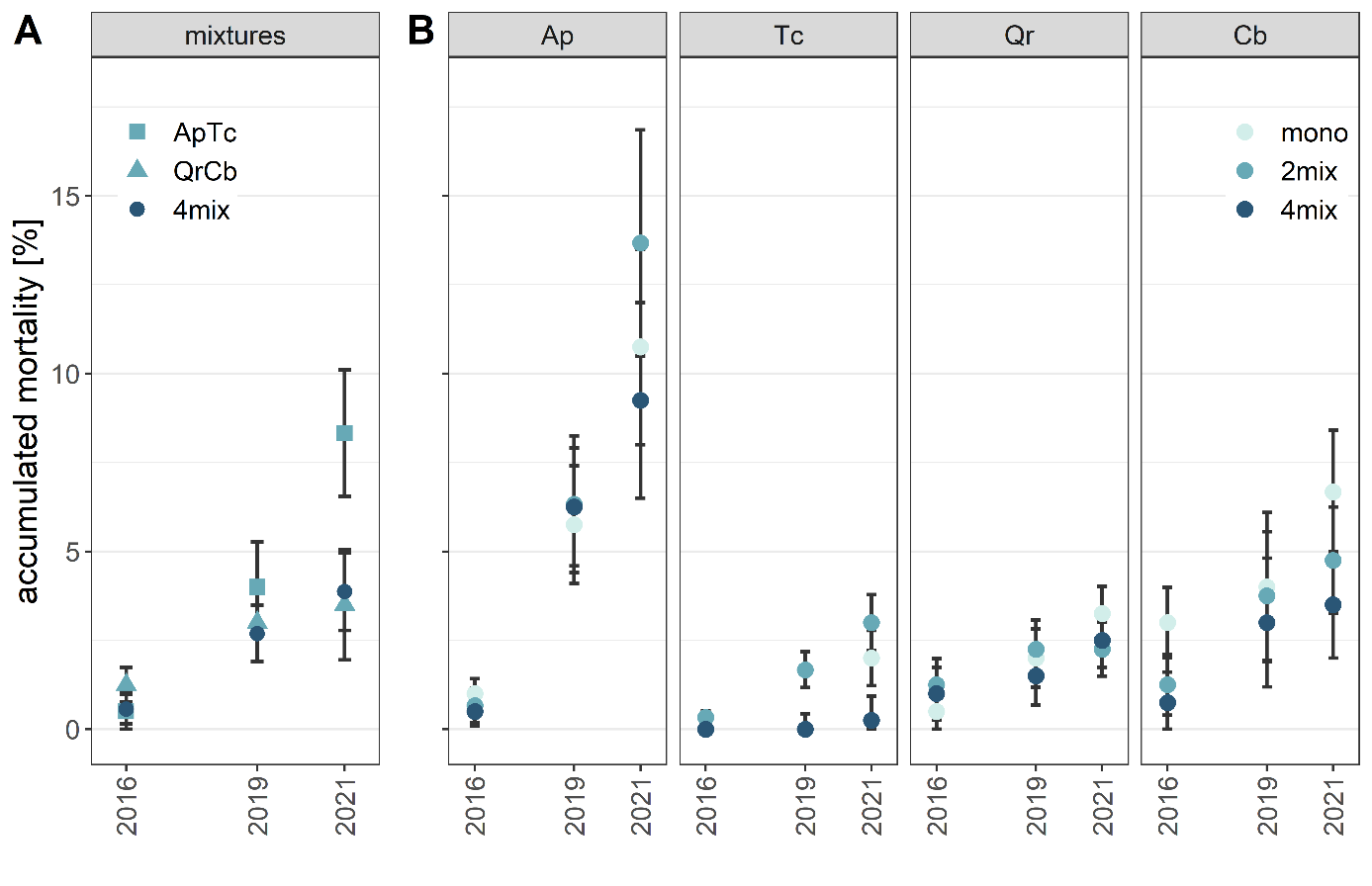


**Fig. S4:** Average, accumulated mortality [%] per **A)** mixture type, and **B)** tree species and diversity level since last replanting in 2014 (LMM with Tukey p-value adjustment; n = 3-4; mean ± SE). Seven plot types: monocultures (*Acer platanoides*, Ap; *Tilia cordata*, Tc; *Quercus robur*, Qr; and *Carpinus betulus*, Cb; ‘mono’), 2-species (ApTc, QrCb; ‘2mix’) and 4-species (‘4mix’) mixtures.


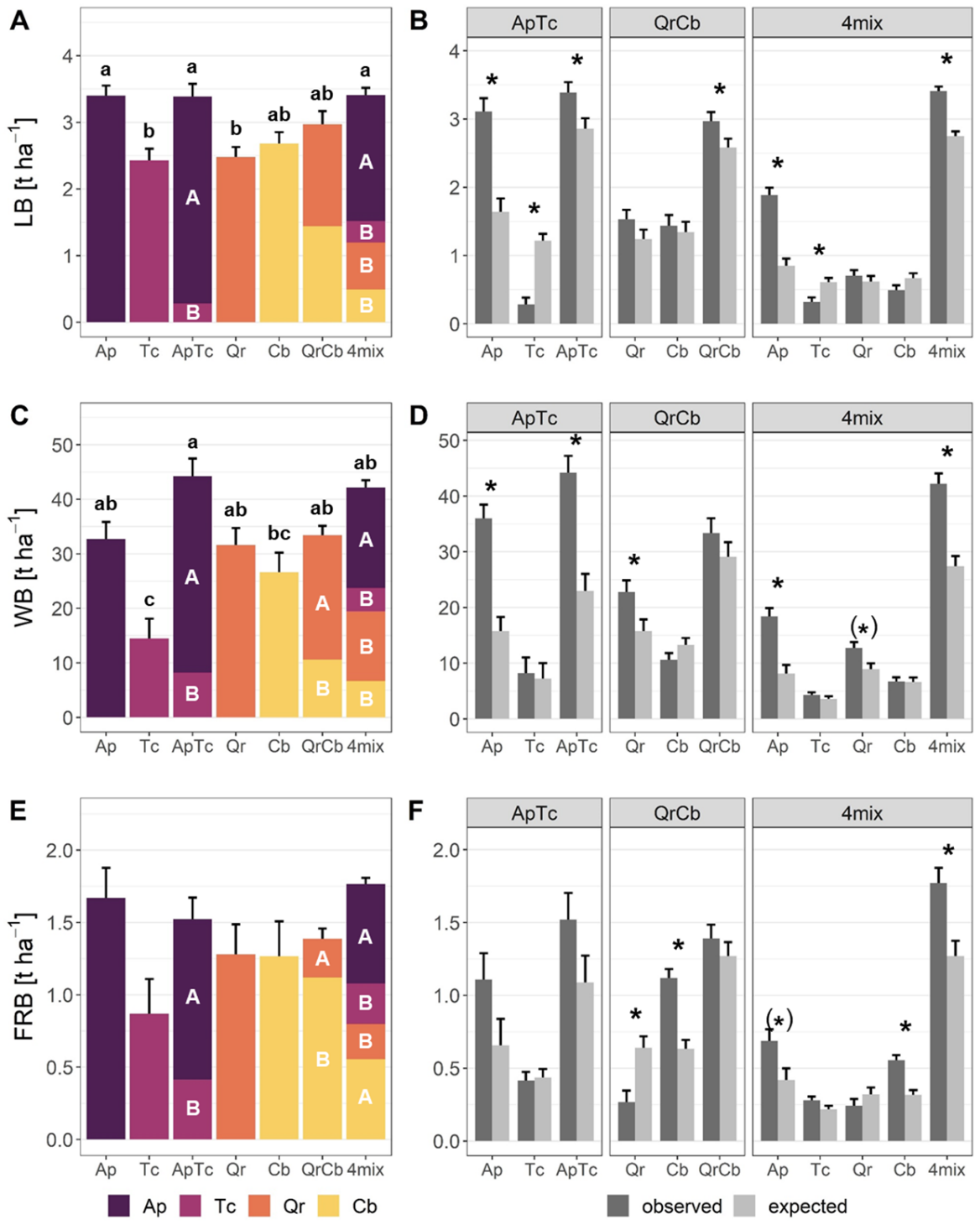


**Fig. S5:** Leaf biomass (LB), wood biomass (WB) and fine root biomass (FRB) of different species and diversity levels. Organ- and species-specific biomass of A) leaf, C) wood and E) fine roots per plot type. Observed (dark grey) and expected (light grey) biomass of the respective organs (B, D, F) of 2- and 4-species mixture plots and their component species. Uppercase letters indicate significant differences between the contribution of each tree species to the total biomass of the respective mixture types. Lowercase letters indicate significant differences (p <0.05; LMM with Tukey p-value adjustment; n = 25; mean ± SE) between plot types. Asterisks indicate significant differences between observed and expected biomasses of mixtures; parentheses denote trends (p <0.1). Tree species monocultures (*Acer platanoides*, Ap; *Tilia cordata*, Tc; *Quercus robur*, Qr, and *Carpinus betulus*, Cb), 2-species mixtures (ApTc, QrCb) and the 4-species mixture (‘4mix’). See Fig. 3 for a simplified version of this figure.


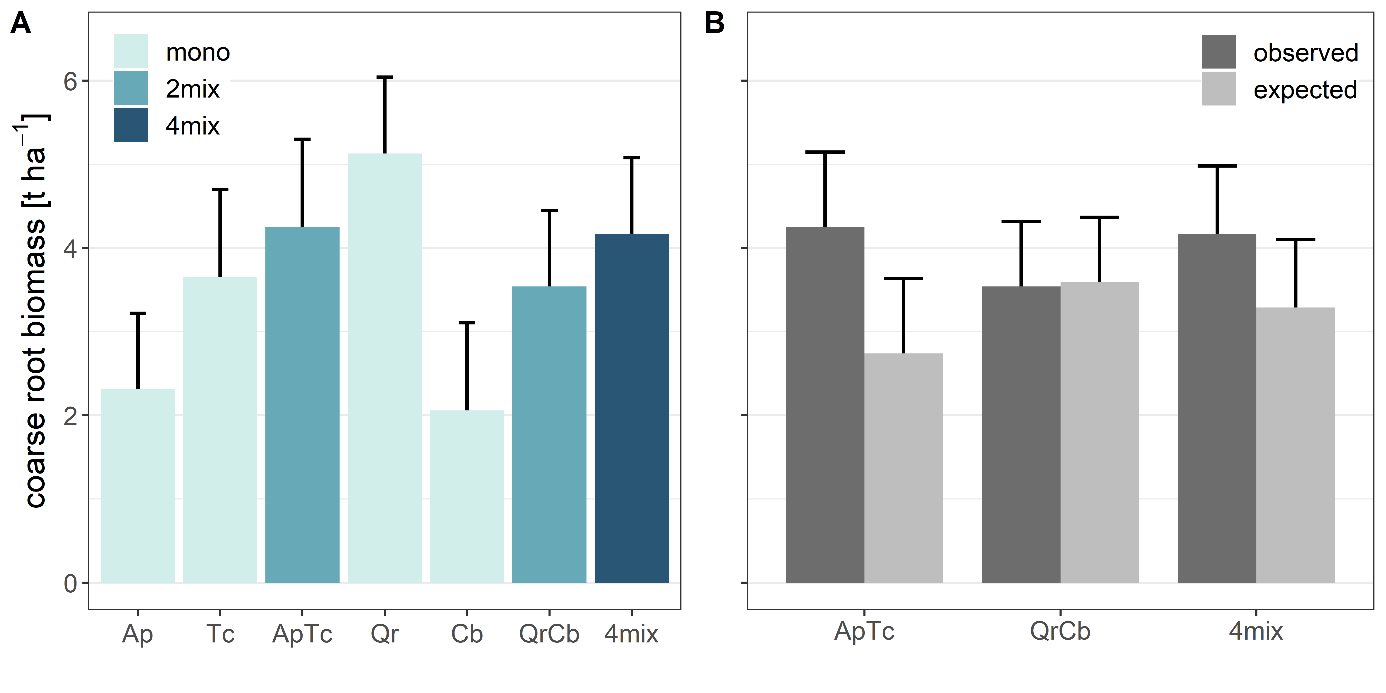


**Fig. S6:** Coarse root biomass (CRB) **A)** per plot type and diversity level. **B)** observed (dark grey) and expected (light grey) CRB per plot type (n = 3-4; mean ± SE). Seven plot types: monocultures (*Acer platanoides*, Ap; *Tilia cordata*, Tc; *Quercus robur*, Qr; and *Carpinus betulus*, Cb; ‘mono’), 2-species (ApTc, QrCb; ‘2mix’) and 4-species (‘4mix’) mixtures.


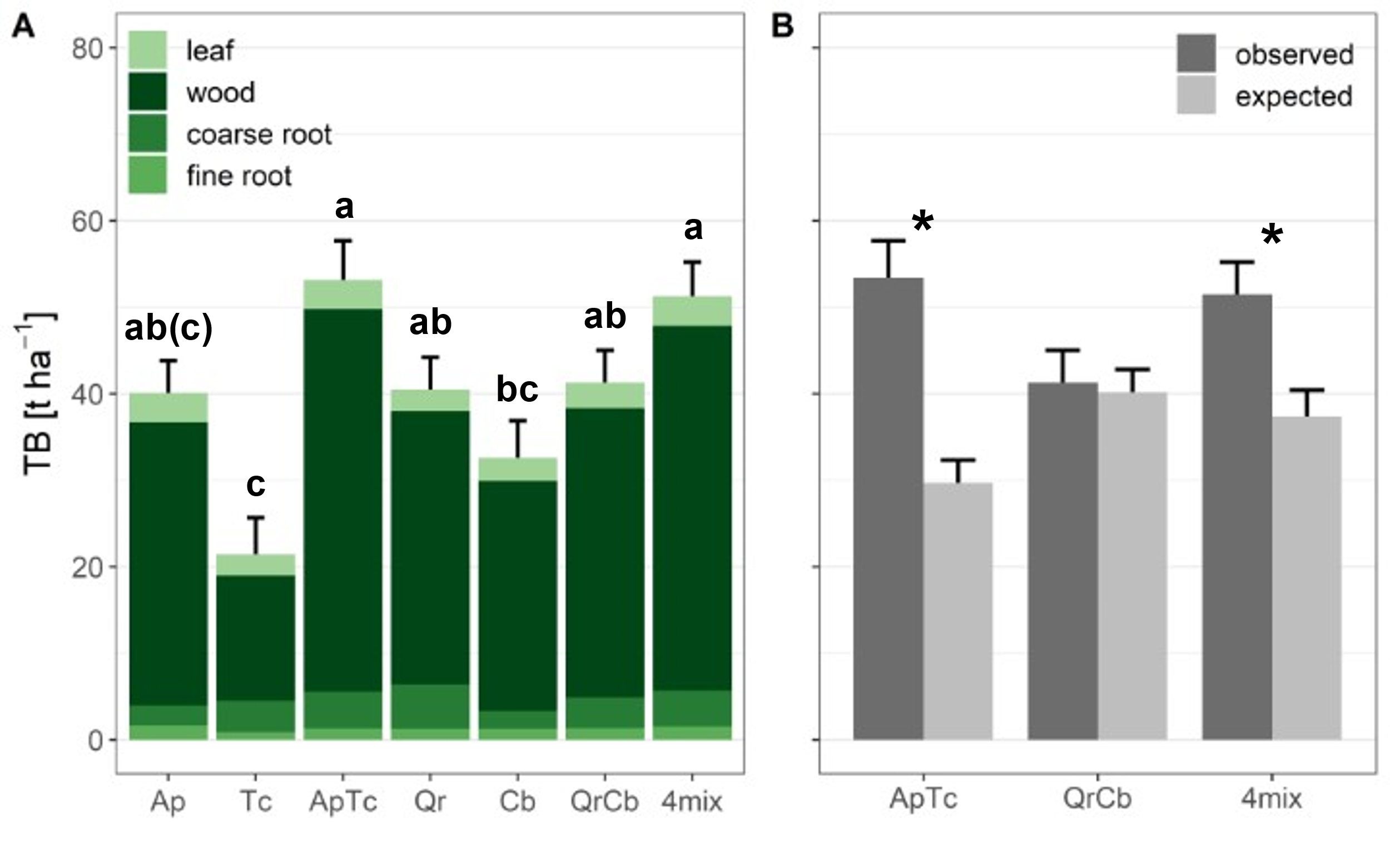


**Fig. S7:** Total plant biomass (TB), calculated as the sum of leaf, wood, coarse root, and fine root biomass, **A)** per plot type, colour-coded by biomass fraction. **B)** observed (dark grey) and expected (light grey) TB per plot type. Seven plot types: monocultures (*Acer platanoides*, Ap; *Tilia cordata*, Tc; *Quercus robur*, Qr; and *Carpinus betulus*, Cb; ‘mono’), 2-species (ApTc, QrCb) and 4-species (‘4mix’) mixtures. Letters indicate significant differences (p <0.05; LMM with Tukey p-value adjustment; n = 3-4; mean ± SE) between the total biomasses. Asterisks indicate significant differences between observed and expected biomass in the respective mixture. Letters in parentheses denote trends (p <0.1).


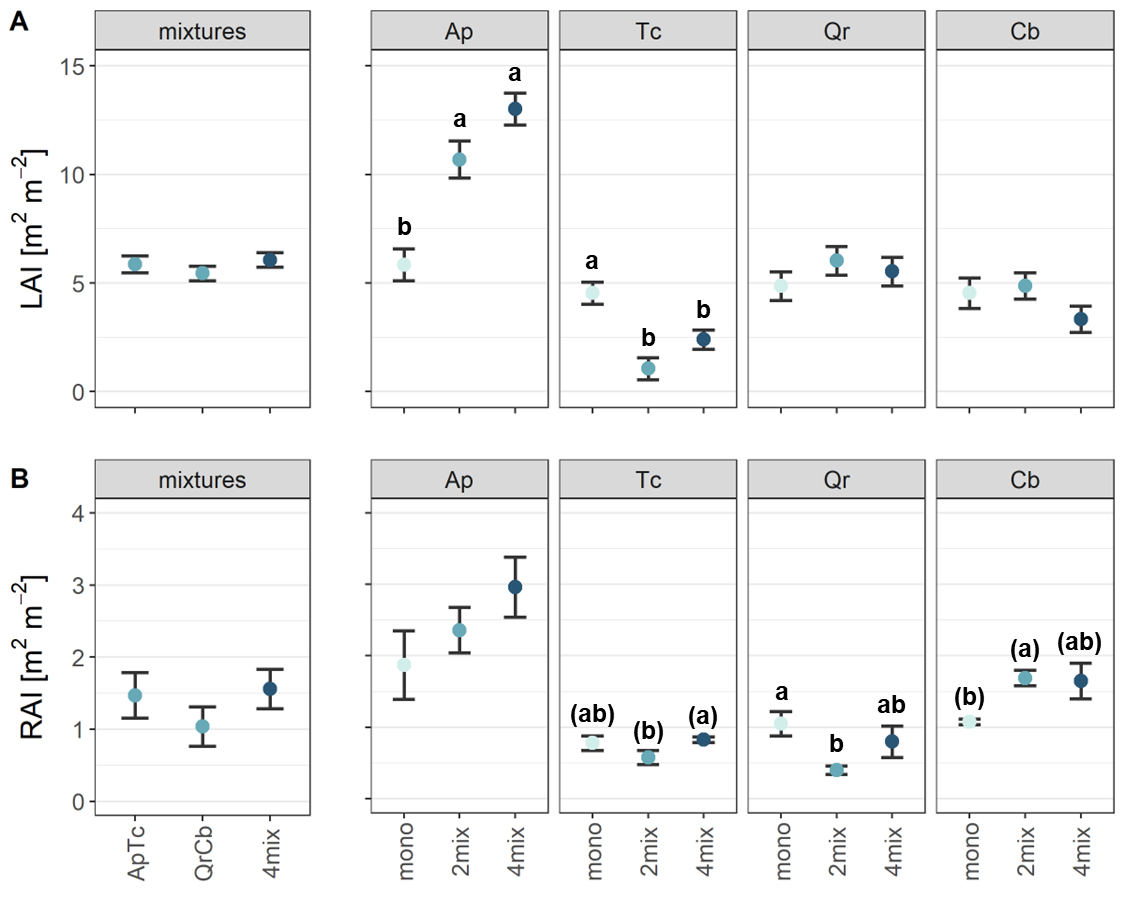


**Fig. S8:** **A)** Leaf area index (LAI) per plot type and LAI_n_ per tree species and diversity level, as well as **B)** fine root area index (RAI) per plot type and RAI_n_ per tree species and diversity level. Seven plot types: monocultures (*Acer platanoides*, Ap; *Tilia cordata*, Tc; *Quercus robur*, Qr; and *Carpinus betulus*, Cb; ‘mono’), 2-species (ApTc, QrCb; ‘2mix’) and 4-species (‘4mix’) mixtures. Letters indicate significant differences (p <0.5; LMM with Tukey p-value adjustment; n = 3-4; mean ± SE), and letters in parentheses denote trends (p <0.1).


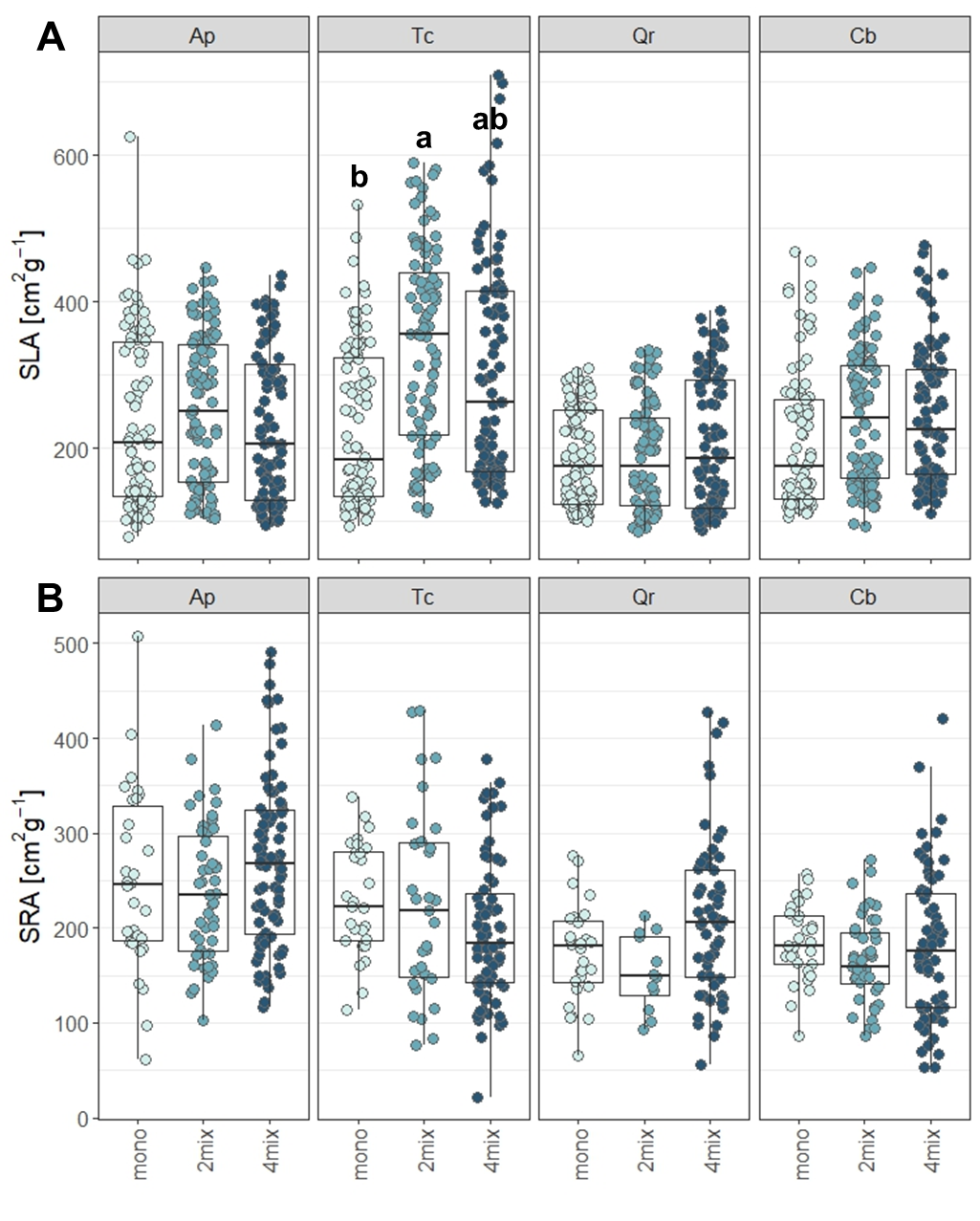


**Fig. S9**: **A)** Specific leaf area (SLA) and **B)** specific root area (SRA) per tree species and diversity level. Species are *Acer platanoides* (Ap), *Tilia cordata* (Tc), *Quercus robur* (Qr), and *Carpinus betulus* (Cb) in monoculture, 2- species (‘2mix’) and 4-species mixture (‘4mix’). Letters indicate significant differences (p <0.05; LMM with Tukey p-value adjustment; n_SLA_ = 255-263, n_SRA_ = 106-156; mean ± SE).


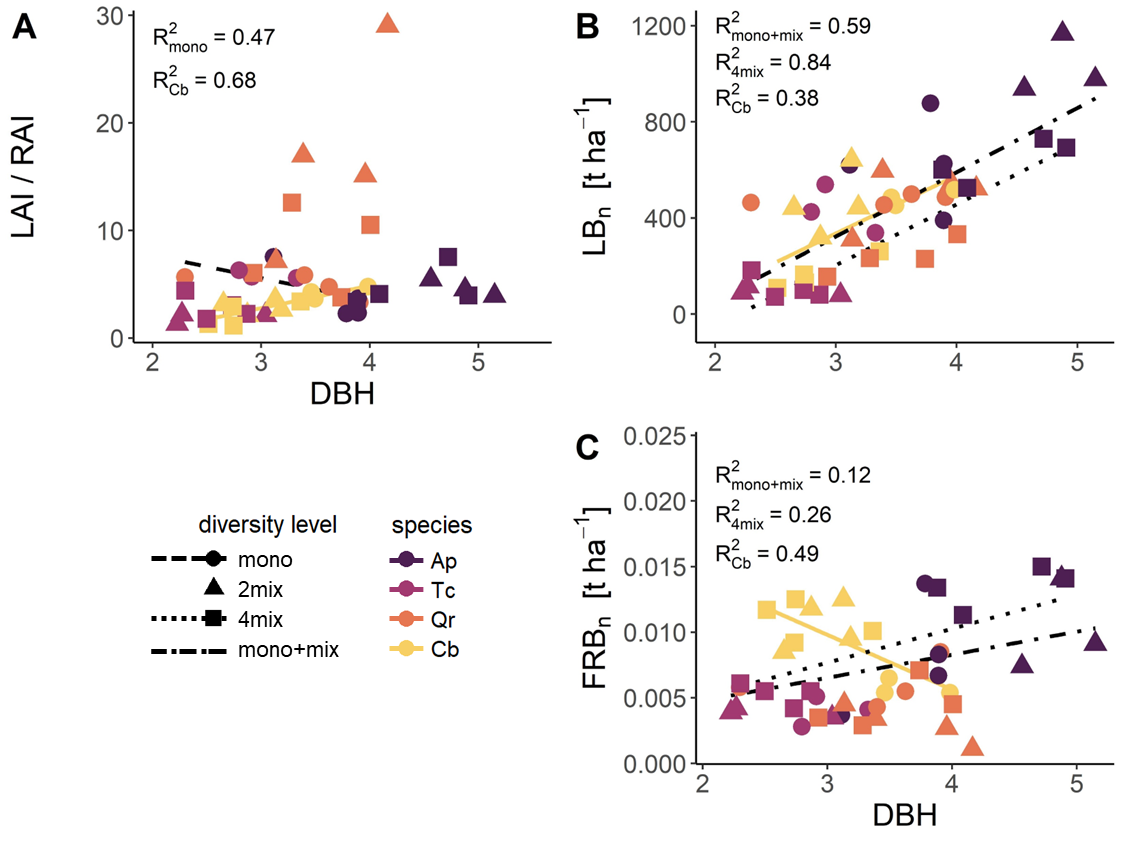


**Fig. S10**: Relationships between tree diameter at breast height (DBH) and **A)** leaf area index to root area index ratio (LAI/RAI), **B)** normalised leaf biomass (LB_n_), and **C)** normalised fine root biomass (FRB_n_) per tree species (Ap = *Acer platanoides*, Tc = *Tilia cordata*, Qr = *Quercus robur*, Cb = *Carpinus betulus*) and diversity levels, i.e. monocultures (circle), 2-species mixtures (‘2mix’; triangle), and 4-species mixtures (4mix; ‘square’). Lines indicate significant (p <0.05) linear relationships per tree species and/or diversity levels; coefficients of determination are given.


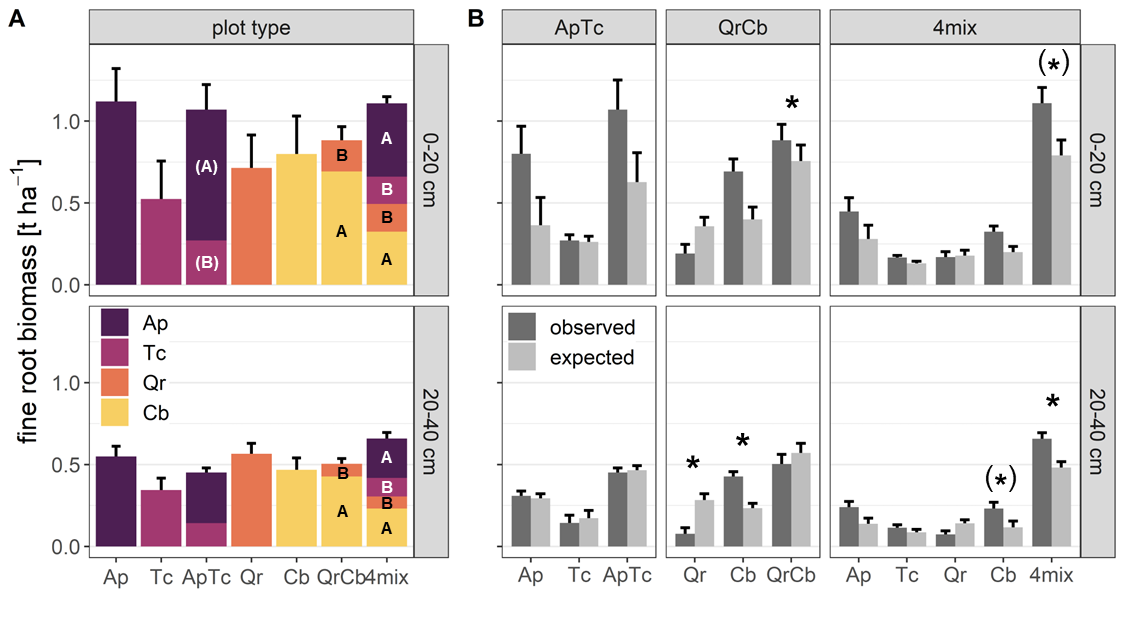


**Fig. S11:** Fine root biomass (FRB) of different species and diversity levels, separated by mineral soil horizon (0-20 cm and 20-40 cm). **A)** Fine root biomass per plot type. **B)** Observed (dark grey) and expected (light grey) fine root biomass of 2- and 4-species mixture plots and their component species. Tree species monocultures (*Acer platanoides*, Ap; *Tilia cordata*, Tc; *Quercus robur*, Qr, and *Carpinus betulus*, Cb), 2-species mixtures (ApTc, QrCb) and the 4-species mixture (‘4mix’). Uppercase letters indicate significant differences between the contribution of each tree species to the total biomass of the respective mixture types. Lowercase letters indicate significant differences (p <0.05; LMM with Tukey p-value adjustment; n = 3-4; mean ± SE) between plot types. Asterisks indicate significant differences between observed and expected biomasses of mixtures; parentheses denote trends (p <0.1).


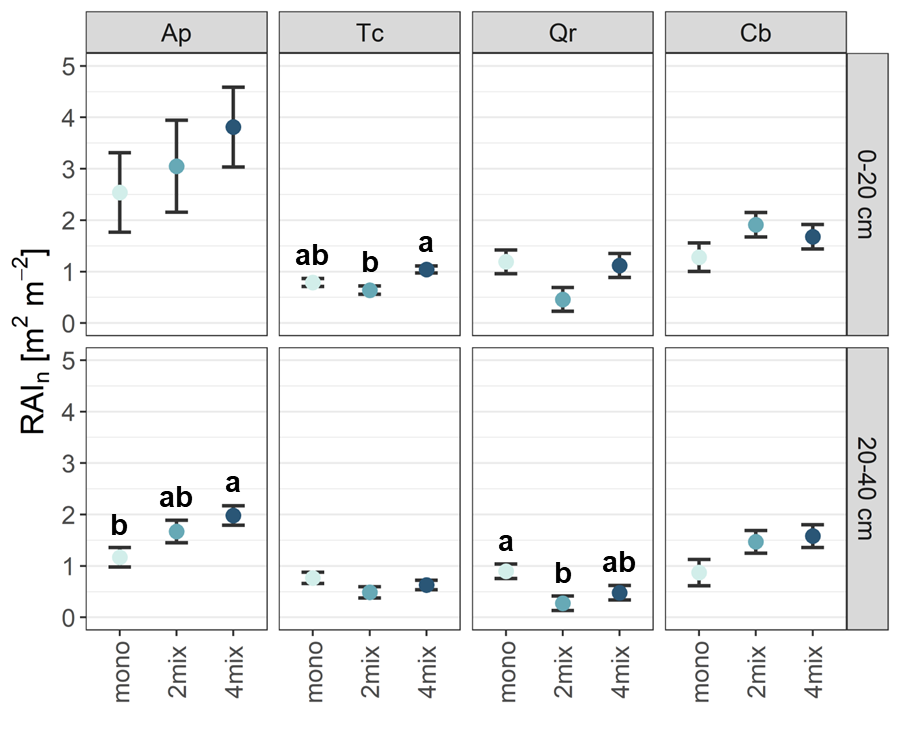


**Fig. S12:** Normalized fine root area index (RAI_n_), separated by mineral soil horizon. Species are *Acer platanoides* (Ap), *Tilia cordata* (Tc), *Quercus robur* (Qr), and *Carpinus betulus* (Cb) in monoculture, 2-species (‘2mix’) and 4-species mixture (‘4mix’). Lowercase letters indicate significant differences (p <0.05; LMM with Tukey p-value adjustment; n = 3-4; mean ± SE).

1. Source: GeoSphere Austria, weather station Langenlebarn [↑](#footnote-ref-2)
2. **IPCC. 2007.** *Climate Change 2007: The Physical Science Basis. Contribution of Working Group I to the Fourth Assessment Report of the Intergovernmental Panel on Climate Change.* Cambridge University Press, Cambridge, United Kingdom and New York, NY, USA [↑](#footnote-ref-3)
