# Supplementary material for: Early overyielding in a mixed deciduous forest is driven by both above- and below-ground species-specific acclimatization
Source: Ann Bot. 2024 Sep 23;134(6):1077–96. doi: 10.1093/aob/mcae150 (PMC11687630; doi:10.1093/aob/mcae150)
Supplement: mcae150_suppl_Supplementary_Materials [file mcae150_suppl_supplementary_materials.zip › aob-24255-s05.docx]

**Supplementary Tables**

**Table S1:** Allometric models for tree species to estimate wood biomass (WB). To examine the relationship between wood biomass and tree parameters, exponential (Y = a × e^bx^), power (Y = a × x^b^) and logarithm (Y = y0 + a ln(x)) models were considered, where x = tree variables, i.e. diameter at breast height (DBH), (DBH)^2^ × height (H), and basal dia. (diameter at 17.5 cm); a, b and y0 are regression coefficients. Equations in bold were considered ‘best fit’ to predict wood biomass.

| **Tree species** | **Variables** | **Equations** | **R^2^** | **RMSE*** | **AICc*** |
| --- | --- | --- | --- | --- | --- |
| *Acer platanoides* | **DBH (cm)** | **Y = 0.1150 x ^2.4078^** | **0.9858** | **0.479340** | **-8.992910** |
|  | (DBH)^2^ H | Y = 0.9645 x ^0.9444^ | 0.9645 | 0.757806 | 0.167555 |
|  | Basal Dia. (cm) | Y = 0.3009 e ^0.4021x^ | 0.8861 | 1.357677 | 11.82981 |
| *Tilia cordata* | DBH (cm) | Y = 0.1396 e^0.6572x^ | 0.9446 | 0.229386 | -23.73230 |
|  | **(DBH)^2^ H** | **Y = 0.6243 e^0.0129x^** | **0.9452** | **0.228117** | **-23.8470** |
|  | Basal Dia. (cm) | Y = 0.0278 x^2.4257^ | 0.9291 | 0.259437 | -21.2702 |
| *Quercus robur* | **DBH (cm)** | **Y = 0.6495 e ^0.4104x^** | **0.9851** | **0.567087** | **-5.63057** |
|  | (DBH)^2^ H | Y = 0.0362 x ^1.0238^ | 0.9758 | 0.723254 | -0.76562 |
|  | Basal Dia. (cm) | Y = 0.2819 e ^0.2887x^ | 0.9710 | 0.791049 | 1.02638 |
| *Carpinus betulus* | DBH (mm) | Y = 0.1558 x^2.1250^ | 0.9200 | 0.345856 | -15.5201 |
|  | (DBH)^2^ H | Y = -5.9387 + 2.1332 ln(x) | 0.8469 | 0.478511 | -9.02736 |
|  | **Basal Dia. (mm)** | **Y = 0.0582 x^2.2435^** | **0.9867** | **0.140896** | **-33.48120** |

*RMSE, root mean square error; AICc, Akaike information criteria (corrected)

**Table S2:** Above- and belowground parameters per diversity level and tree species: *Acer platanoides* (Ap), *Tilia cordata* (Tc), *Quercus robur* (Qr), and *Carpinus betulus* (Cb), in monocultures ('mono’), 2-species ( ‘2mix’) and 4-species (‘4mix’) mixtures. WB = wood biomass (i.e., stem and branches), LB = leaf biomass, CRB = coarse root biomass (measured only on plot-level), FRB = fine root biomass (0-40 cm), LAI = leaf area index, RAI = root area index, (h)MCD = (height of) maximum crown diameter, hFB = height of first branch, BA_130_ = tree basal area at 130 cm height (mean ± SE). Mortality as accumulated between 2014 and 2021.

| **Diversity**  **level** | **Tree**  **species** | **Biomass** | | | | **Traits** | | | | | **Stem dimensions and Mortality** | | |
| --- | --- | --- | --- | --- | --- | --- | --- | --- | --- | --- | --- | --- | --- |
|  |  | **WB** | **LB** | **CRB** | **FRB** | **LAI** | **RAI** | **hMCD** | **hFB** | **MCD** | **Height** | **BA_130_** | **Mortality** |
|  |  | **[t ha^-1^]** | **[t ha^-1^]** | **[t ha^-1^]** | **[t ha^-1^]** | **[m^2^ m^-2^]** | **[m^2^ m^-2^]** | **[m]** | **[m]** | **[m]** | **[m]** | **[cm^2^]** | **[%]** |
| mono | Ap | 32.7 ± 3.1 | 3.4 ± 0.2 | 2.3 ± 0.7 | 1.7 ± 0.2 | 5.9 ± 0.7 | 1.9 ± 0.5 | 3.9 ± 0.2 | 1.5 ± 0.2 | 1.3 ± 0.1 | 5.8 ± 0.3 | 23.5 ± 2.1 | 10.7 ± 2.7 |
| mono | Tc | 14.5 ± 3.6 | 2.4 ± 0.2 | 3.7 ± 1.1 | 0.9 ± 0.2 | 4.5 ± 0.5 | 0.8 ± 0.1 | 2.5 ± 0.3 | 0.7 ± 0.1 | 1.3 ± 0.1 | 3.9 ± 0.3 | 18.7 ± 1.1 | 2.0 ± 0.8 |
| mono | Qr | 31.6 ± 3.1 | 2.5 ± 0.2 | 5.1 ± 1.2 | 1.3 ± 0.2 | 4.9 ± 0.7 | 1.1 ± 0.2 | 3.0 ± 0.3 | 1.8 ± 0.3 | 1.4 ± 0.1 | 4.2 ± 0.3 | 19.0 ± 2.5 | 3.3 ± 0.8 |
| mono | Cb | 26.6 ± 3.6 | 2.7 ± 0.2 | 2.1 ± 0.5 | 1.3 ± 0.2 | 4.5 ± 0.7 | 1.1 ± 0.04 | 3.5 ± 0.2 | 1.2 ± 0.2 | 1.5 ± 0.1 | 4.8 ± 0.2 | 21.2 ± 2.0 | 6.7 ± 1.7 |
| 2mix | Ap | 36.0 ± 2.5 | 3.1 ± 0.2 | 4.3 ± 0.9 | 1.1 ± 0.2 | 5.4 ± 0.5 | 1.2 ± 0.2 | 4.4 ± 0.3 | 1.7 ± 0.2 | 1.7 ± 0.1 | 6.6 ± 0.3 | 40.1 ± 2.4 | 13.7 ± 3.2 |
| 2mix | Tc | 8.3 ± 3.9 | 0.3 ± 0.2 |  | 0.4 ± 0.2 | 0.5 ± 0.1 | 0.3 ± 0.1 | 3.2 ± 0.2 | 1.1 ± 0.1 | 1.5 ± 0.1 | 4.1 ± 0.3 | 12.5 ± 1.1 | 3.0 ± 0.8 |
| 2mix | Qr | 22.8 ± 1.7 | 1.5 ± 0.2 | 3.6 ± 0.8 | 0.3 ± 0.1 | 3.0 ± 0.4 | 0.2 ± 0.0 | 3.2 ± 0.3 | 1.4 ± 0.3 | 1.6 ± 0.1 | 4.5 ± 0.3 | 22.3 ± 2.5 | 2.3 ± 0.8 |
| 2mix | Cb | 10.6 ± 1.7 | 1.4 ± 0.2 |  | 1.1 ± 0.1 | 2.4 ± 0.4 | 0.8 ± 0.1 | 2.5 ± 0.1 | 1.0 ± 0.2 | 1.6 ± 0.1 | 4.2 ± 0.2 | 15.2 ± 1.8 | 4.8 ± 1.5 |
| 4mix | Ap | 18.4 ± 1.3 | 1.9 ± 0.1 | 4.2 ± 0.8 | 0.7 ± 0.04 | 3.3 ± 0.2 | 0.7 ± 0.1 | 3.6 ± 0.2 | 1.4 ± 0.2 | 1.5 ± 0.1 | 5.7 ± 0.3 | 33.7 ± 2.2 | 9.3 ± 2.8 |
| 4mix | Tc | 4.3 ± 1.3 | 0.3 ± 0.1 |  | 0.3 ± 0.04 | 0.6 ± 0.1 | 0.2 ± 0.0 | 2.4 ± 0.2 | 0.8 ± 0.1 | 1.4 ± 0.1 | 3.8 ± 0.3 | 14.4 ± 1.0 | 0.3 ± 0.7 |
| 4mix | Qr | 12.8 ± 1.3 | 0.7 ± 0.1 |  | 0.2 ± 0.04 | 1.4 ± 0.2 | 0.2 ± 0.1 | 2.9 ± 0.3 | 1.7 ± 0.3 | 1.4 ± 0.1 | 4.4 ± 0.3 | 21.6 ± 2.5 | 2.5 ± 0.8 |
| 4mix | Cb | 6.7 ± 1.3 | 0.5 ± 0.1 |  | 0.6 ± 0.04 | 0.8 ± 0.2 | 0.4 ± 0.1 | 2.6 ± 0.1 | 0.9 ± 0.2 | 1.5 ± 0.1 | 4.1 ± 0.2 | 14.9 ± 1.8 | 3.5 ± 1.5 |

**Table S3:** Organ-specific mass fractions per diversity level and component species. LMF = leaf mass fraction, SMF = stem mass fraction, RMF = fine root mass fraction (mean ± SE). *Acer platanoides* (Ap), *Tilia cordata* (Tc), *Quercus robur* (Qr), and *Carpinus betulus* (Cb), in monocultures ('mono’), 2-species (ApTc, QrCb; ‘2mix’) and 4-species (‘4mix’) mixtures. See Fig. 4 for significant effects between diversity levels per species.

| **Diversity**  **level** | **Tree**  **species** | **LMF**  **[%]** | **SMF**  **[%]** | **RMF**  **[%]** |
| --- | --- | --- | --- | --- |
| mono | Ap | 9.4 ± 0.9 | 86.3 ± 1.1 | 4.3 ± 0.6 |
| mono | Tc | 14.0 ± 2.2 | 81.1 ± 3.3 | 4.9 ± 1.3 |
| mono | Qr | 7.3 ± 0.6 | 88.9 ± 0.8 | 3.8 ± 0.4 |
| mono | Cb | 8.9 ± 0.5 | 86.9 ± 1.3 | 4.2 ± 1.3 |
| 2mix / ApTc | Ap | 7.8 ± 1.0 | 89.5 ± 1.2 | 2.8 ± 0.6 |
| 2mix / ApTc | Tc | 4.5 ± 2.2 | 89.3 ± 3.3 | 6.2 ± 1.3 |
| 2mix / QrCb | Qr | 6.3 ± 0.6 | 92.6 ± 0.8 | 1.1 ± 0.4 |
| 2mix / QrCb | Cb | 10.9 ± 0.5 | 80.4 ± 1.1 | 8.8 ± 1.2 |
| 4mix | Ap | 9.1 ± 0.9 | 87.6 ± 1.1 | 3.3 ± 0.6 |
| 4mix | Tc | 7.0 ± 1.9 | 87.0 ± 2.9 | 6.0 ± 1.1 |
| 4mix | Qr | 5.1 ± 0.6 | 93.1 ± 0.8 | 1.8 ± 0.4 |
| 4mix | Cb | 6.3 ± 0.5 | 85.9 ± 1.1 | 7.8 ± 1.2 |

**Table S4:** Frequency distribution of species-specific fine root contributions to the total fine root biomass per soil core. Contributions are categorized by comparison to expected values: 'as expected' denotes a contribution within 33% or 66% for 2-species mixtures (ApTc or QrCb, respectively), and 33% or no contribution (e.g. for Ap in CbQrTc) for 4-species mixtures (4mix; see Fig. 1D). Contributions falling outside these ranges are labelled 'less frequent than expected' or 'more frequent than expected'. The 'presence without being in the immediate vicinity' category records instances where roots from a non-included species were found (e.g., Ap in CbQrTc). Percentages reflect the relative frequency of each contribution category within the diversity levels and tree species combinations. Tree species are *Acer platanoides* (Ap), *Tilia cordata* (Tc), *Quercus robur* (Qr), and *Carpinus betulus* (Cb), studied in both 2-species (ApTc, QrCb) and 4-species mixtures (‘4mix’). n_ApTc/QrCb_ = 48, n_4mix_ = 96.

| **Diversity**  **level** | **Tree**  **species** | **Frequency of contribution to soil core [%]** | | | |
| --- | --- | --- | --- | --- | --- |
|  |  | **less frequent than expected** | **as expected** | **more frequent than expected** | **presence without being in the immediate vicinity (triplet)** |
| ApTc | Ap | 8 | 4 | 88 | - |
|  | Tc | 85 | 2 | 13 | - |
| QrCb | Qr | 96 | - | 4 | - |
|  | Cb | 4 | - | 96 | - |
| 4mix | Ap | 23 | 14 | 42 | 22 |
|  | Tc | 51 | 25 | 18 | 6 |
|  | Qr | 54 | 19 | 16 | 12 |
|  | Cb | 44 | 13 | 33 | 10 |
